# The Haematopoietically-expressed homeobox transcription factor: roles in development, physiology and disease

**DOI:** 10.3389/fimmu.2023.1197490

**Published:** 2023-06-16

**Authors:** Jacob T. Jackson, Stephen L. Nutt, Matthew P. McCormack

**Affiliations:** ^1^ Immunology Division, The Walter and Eliza Hall Institute of Medical Research, Parkville, VIC, Australia; ^2^ Department of Medical Biology, University of Melbourne, Parkville, VIC, Australia; ^3^ The Australian Centre for Blood Diseases, Monash University, Melbourne, VIC, Australia; ^4^ iCamuno Biotherapeutics, Melbourne, VIC, Australia

**Keywords:** transcription factor, Hhex, diabetes, haematopoiesis, leakamia, AML, T-ALL

## Abstract

The Haematopoietically expressed homeobox transcription factor (Hhex) is a transcriptional repressor that is of fundamental importance across species, as evident by its evolutionary conservation spanning fish, amphibians, birds, mice and humans. Indeed, Hhex maintains its vital functions throughout the lifespan of the organism, beginning in the oocyte, through fundamental stages of embryogenesis in the foregut endoderm. The endodermal development driven by Hhex gives rise to endocrine organs such as the pancreas in a process which is likely linked to its role as a risk factor in diabetes and pancreatic disorders. Hhex is also required for the normal development of the bile duct and liver, the latter also importantly being the initial site of haematopoiesis. These haematopoietic origins are governed by Hhex, leading to its crucial later roles in definitive haematopoietic stem cell (HSC) self-renewal, lymphopoiesis and haematological malignancy. Hhex is also necessary for the developing forebrain and thyroid gland, with this reliance on Hhex evident in its role in endocrine disorders later in life including a potential role in Alzheimer’s disease. Thus, the roles of Hhex in embryological development throughout evolution appear to be linked to its later roles in a variety of disease processes.

## 1 Background

**Figure 1:**
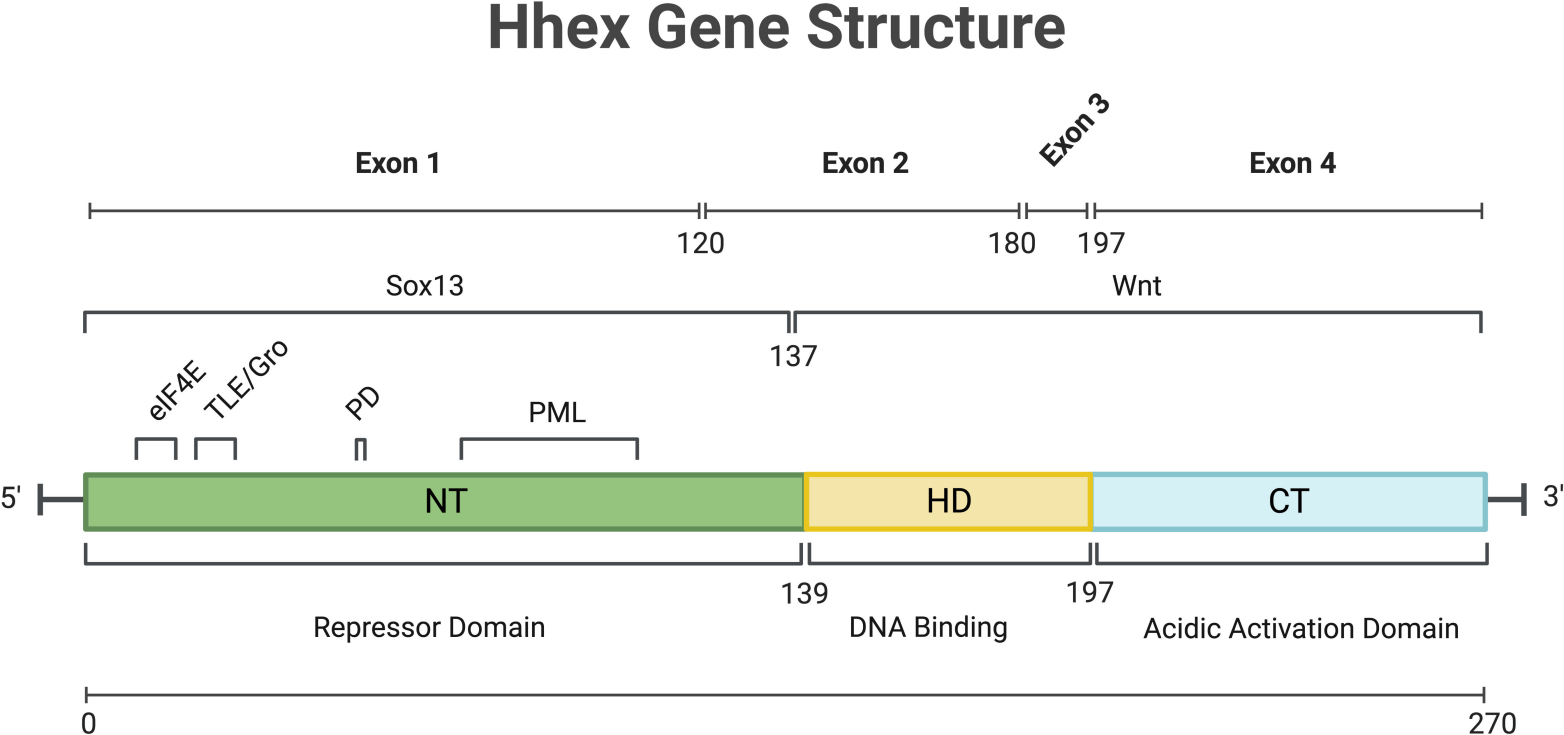
Overview of Hhex gene structure in humans. Numbers indicate amino acid position. C-Terminal (CT), eukaryotic Initiation Factor-4A (eIF4a), Homeo-Domain (HD), N-Terminal (NT), Phosphorylation Domain (PD), Promyelocytic Leukaemia protein (PML), SRY-Box Transcription Factor 13 (Sox13), Transducin-Like Enchancer/Groucho (TLE/Gro), Wingless/Integrated Signalling domain (Wnt). Gene and protein sequence information was obtained from NCBI (NM_002729.5 and NP_002720.1 respectively). Created with BioRender.com.

The Haematopoietically expressed homeobox gene (Hhex), also known as Hex, Xhex in *Xenopus* and Prh (proline rich homeodomain), was first identified in chicken haematopoietic cells, as well as cells of the liver and lungs, with homologues noted in chickens, Xenopus, mice and humans (1–5). Hhex is a non-clustered/divergent/orphan homeobox gene, members of which are distinct from the clustered (Hox) homeobox genes, in that they are spread throughout the genome. The genomic structure of human Hhex was shown to comprise 4 exons (**Figure 1**) located on chromosome 10 whilst in mice Hhex is located on chromosome 19 (2, 5). The Jayaraman laboratory first showed a role for Hhex in haematopoiesis using chicken cells at a similar developmental state to that of megakaryocytic-erythroid progenitors (MEPs). Transformation of these cells by Hhex, specifically the myeloblasts, induced them to proliferate *in vitro (* 6). Early analysis of haematopoietic cell lines at various stages of differentiation quickly revealed Hhex was weakly expressed in T cells and plasma cells, but abundant in developing B cells (7). Hhex is also found in myeloid and osteoclastic progenitors along with MEPs, but downregulated during differentiation (7).

The first study of Hhex noted the DNA binding capacity of the homeodomain and thus its potential for transcriptional regulation (1). Depending on the context and cofactor interactions, Hhex can act as a transcriptional repressor or activator (8, 9). The activation domain of Hhex, regardless of cell type, was determined to be at the carboxy-terminus (10), while the N-terminus of Hhex may be responsible for inhibiting DNA binding by the homeodomain and that may also enable Hhex to form oligomers within the nucleus to mediate its function as discussed further below (**Figure 1**) (11, 12).

As a key regulator of development and haematopoiesis, expression of Hhex must be tightly controlled, and its role as a repressor is key to its utility in haematopoietic stem and progenitor cells. In a study employing murine haematopoietic cell lines it was shown that Hhex was regulated by an element in its first intron (13). This haematopoietic-specific enhancer is bound by GATA-1, GATA-2 and c-Myb (13, 14). Hhex was also identified as a GATA-binding partner in human endothelial cells where its expression is induced by transforming growth factor (TGF)-β1 with Hhex then driving Flk-1 expression and downregulating vascular endothelial growth factor (VEGF) signalling (15).

Following translation, Hhex protein is regulated by and interacts with a number of proteins in undertaking its functions. In humans, oligomers of Hhex, in the form of octamers, have been shown to bind with high affinity to numerous locations within the promoter of *Goosecoid* and the DNA is wrapped by Hhex binding to promote transcriptional repression (16). These oligomeric forms of Hhex are highly stable, resisting both chemical and thermal denaturing (17, 18). Hhex also regulates the retention of Groucho/Transducin-like enhancer protein (TLE) proteins in the nucleus *via* direct binding, and this Hhex/TLE interaction is important for transcriptional repression (**Figure 1**) (19).

It also was reported that Hhex bound Jun *via* helix III of the Hhex homeodomain implying a role of Hhex in cytokine/growth factor signalling (20). In a haematopoietic cell line, K562, the N-terminal proline-rich domain of Hhex was observed to interact with the proteasome, specifically the HC8 subunit within the 20S and 26S proteasomes (21). Whilst Hhex was cleaved slowly by the proteasome, this process was not required for the transcriptional repression mediated by Hhex (21). Truncated forms of Hhex, formed subsequent to the proteolysis process, were still able to bind DNA (21). Hhex can be phosphorylated by the β subunit of CK2 at residues S163 and S177, an event that inhibits DNA binding by Hhex, which in turn is reversible by dephosphorylation (22). In human U937 cells, Hhex was reported as a potential negative regulator of eukaryotic translation initiation factor 4E (eIF4E) in myeloid cells (23). In this context, Hhex was thought to regulate cellular translation by inhibiting eIF4E-dependent Cyclin D1 mRNA transport (23). HOXA9 was required for eIF4E function, which in turn competes with Hhex as a functional repressor of eIF4E, and if dysregulated can lead to leukemogenesis (24). Moreover, eIF4E-dependent nuclear export of Cyclin D1 and ornithine decarboxylase mRNAs is stimulated by HOXA9 (24).

Together, these results clearly demonstrate Hhex regulates and is regulated by diverse intracellular processes depending on the cellular context and warrant further research to fully understand the post-translational roles of Hhex in diverse cell types.

## 2 Role of Hhex in embryogenesis

**Figure 2:**
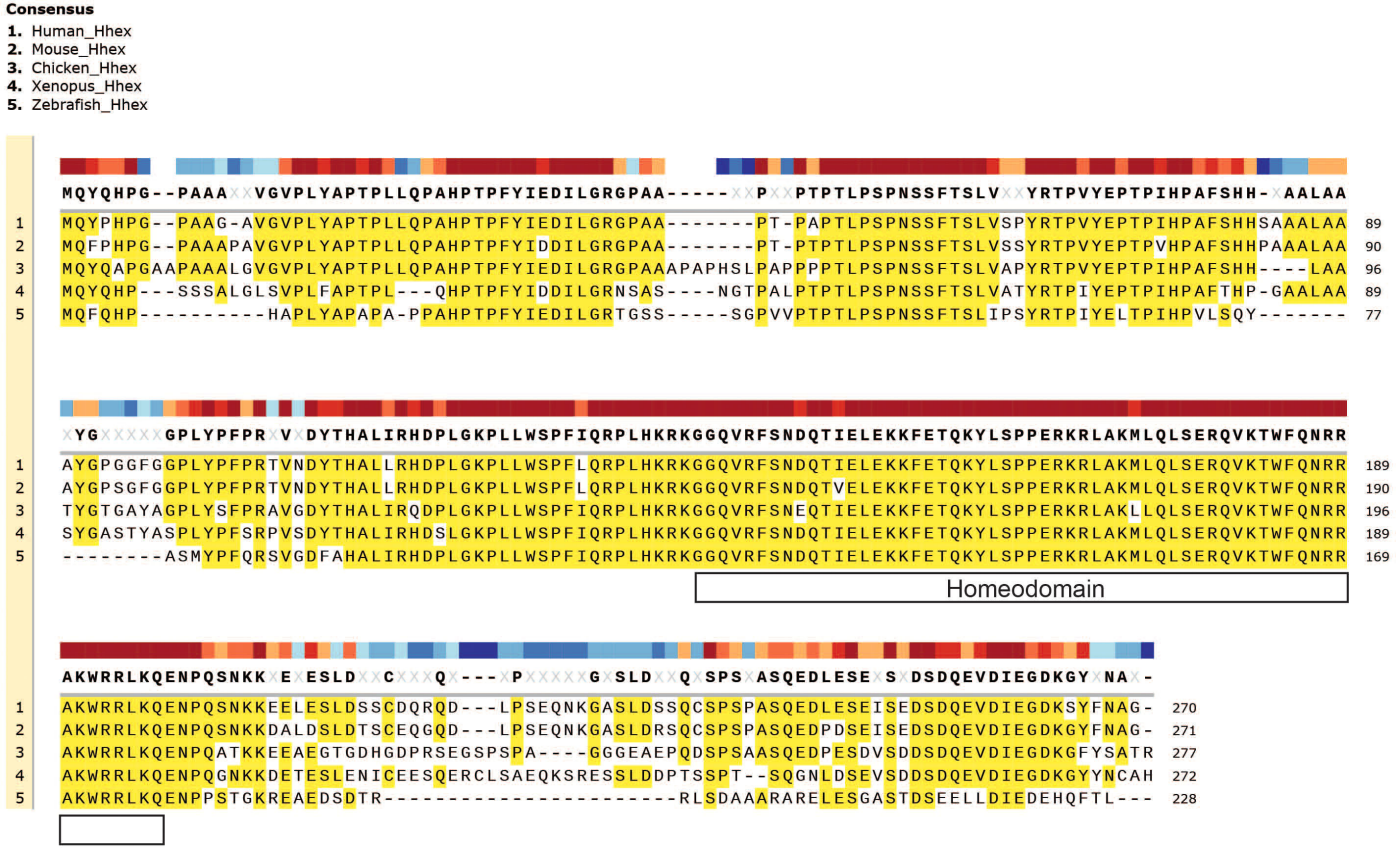
Evolutionary conservation of Hhex between divergent species. Gene amino acid sequence alignment of Hhex between species including Human, Mouse, Chicken, Xenopus and Zebrafish. Amino acid alignment highlighted in yellow. The topmost, colour-coded bar indicates the degree of conservation between species. Gene sequences were obtained from UniProt and aligned with MUSCLE alignment using SnapGene software (Version 5.2.4).

Hhex plays a fundamental role in embryogenesis in many organisms throughout evolution including in fish, amphibians, birds, mice and humans, demonstrating its highly evolutionarily conserved role in vertebrate development, which is also strikingly revealed by amino acid sequence alignment, particularly with regards to the homeodomain (**Figure 2**).

### 2.1 Humans

**Figure 3:**
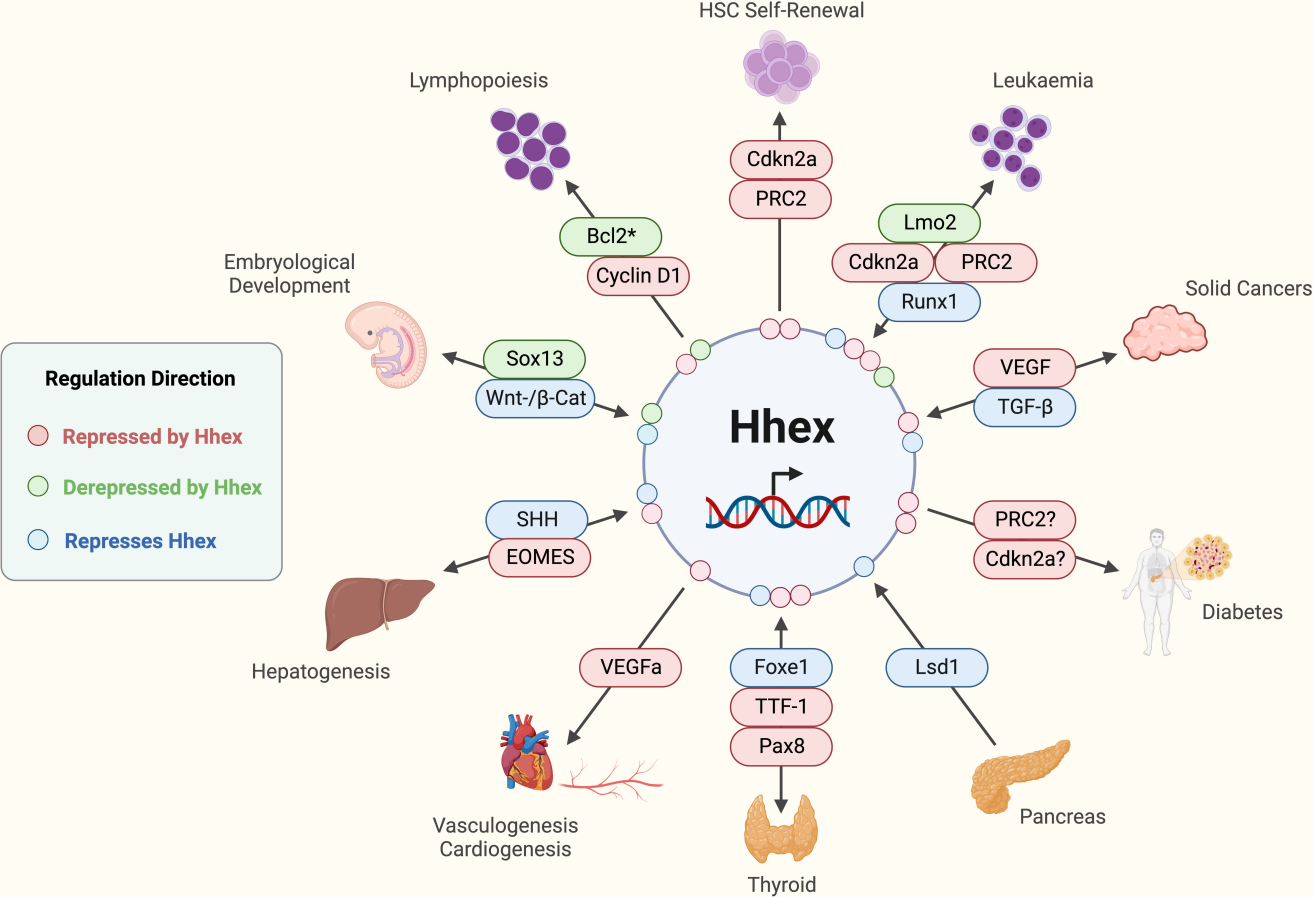
Established functions and interactions of Hhex in mammalian cells in various tissue and disease contexts. *Potentially other anti-apoptotic molecules are involved depending on the immunological cell type in question. Acute Myeloid Leukaemia (AML), B-cell lymphoma 2 (Bcl2), Cyclin-dependent kinase 2a (Cdkn2a), Eomesodermin (EOMES), Forkhead box e1 (Foxe1), Haematopoietic Stem Cell (HSC), (Pax8), Lim domain only 2 (Lmo2), Lysine-specific demethylase 1 (Lsd1), Polycomb Repressive Complex 2 (PRC2), Runt-related transcription factor 1 (Runx1), Sonic Hedgehog (SHH), SRY-box transcription factor 13 (Sox13), T cell Acute Lymphoblastic Leukaemia (T-ALL), Tumour Growth Factor-beta (TGF-β), Thyroid Transcription Factor-1 (TTF-1), Vascular Endothelial Growth Factor (VEGF), Wingless-Integrated/β-Catenin (Wnt/β-Cat). Created with BioRender.com.

Hhex expression was detected in a human cDNA library of oocytes and embryos (up to 10 weeks old) along with other Hox family genes including HOXD8, HOXD1 and OCT1, as well as HOXA7 exclusively in oocytes (25). In human ESCs and inducible pluripotent stem cells (iPSCs), Hhex overexpression was able to induce hepatoblasts (26). The same group also showed that Hhex was driving hepatogenesis through repression of eomesdermin (EOMES) expression (**Figure 3**) (27). Moreover, in human iPSCs, Hhex, and its closely related orphan homeobox gene Hlx, enhance early-phase reprogramming, whilst blocking pluripotency in somatic cells (28). Hhex expression was also found to be restricted by Sonic Hedgehog (Shh) activity in a human ES model of pancreatic development where Hhex was one of several epithelium markers, along with HNFα, Pax6 and PTF1α to be downregulated by Shh (**Figure 3**) (29). In a recent study, Hhex was demonstrated to be a “gatekeeper” of pancreatic development in human IPSCs, with its deletion resulting in liver and duodenum development (30). This commitment to pancreatic development driven by Hhex was observed in combination with other transcription factors including FOXA1, FOXA2 and GATA4 (30). Additionally, inhibition of all-trans retinoic acid was also noted to downregulate HHEX in a pancreatic endoderm model using hESCs (31). Whilst there is a clear importance of Hhex demonstrated in human embryological development, much of what we understand regarding its key developmental roles has nevertheless been extensively gleaned from murine studies as detailed below.

### 2.2 Mice

Hhex was initially observed as exhibiting endodermal expression, marking developing liver and foregut, as well as mesodermal expression with nascent blood islands in the visceral yolk sac of murine embryos (32). Further characterisation of the murine embryo by *in situ* hybridisation revealed Hhex was expressed in the chorion of the ectoplacental cavity and weakly in the visceral endoderm of the future yolk sac at E7.5, in liver and thyroid tissues only at E9.5 and in the foetal liver, lung and thyroid at E12.5-15.5 (33). Hhex was further shown to be essential for liver formation in the murine embryo at day E9.5 (33). As well as being important for thyroid and lung, Hhex Knock Out (KO) mice revealed that Hhex was involved in the hepatic ectoderm, as well as a role in monopoiesis, with embryonic lethality ultimately resulting by E10.5 (33). Bogue, et al. showed Hhex plays a role in murine foregut organogenesis including the thymus, where Hhex is downregulated by E18.5 (34). Whilst the specification of thyroid cells does not require Hhex (35), it is nevertheless required for normal thyroid development, where by E10 it is the organ with the highest Hhex expression, with its expression remaining high until E18.5 (34). Additionally, the absence of Pax8 failed to affect the expression of Hhex in the developing thyroid at E9, but Hhex was undetectable at E10, suggesting that Pax8 is required for maintaining Hhex expression, but not the induction of its expression (35). These studies collectively point to later roles for Hhex in endocrinology. It was also shown that Hhex is vital for developing lung, bile duct, gall bladder and pancreas. High, and essential, expression of Hhex in the developing liver endoderm was also reported, potentially linking to Hhex’s vital importance in additionally providing the necessary organ environment to facilitate haematopoiesis, detailed further in subsequent sections of this review (34, 36).

Further characterisation of Hhex’s role in endodermal development within the embryo showed that in the absence of Hhex-anterior visceral endoderm (AVE) repression, Bmp2 is not present in the proximal visceral endoderm and Wingless/Integrated 3 (Wnt3) and Nodal are not properly limited to the posterior epiblast (37). Hhex-AVE null embryos then exhibited later initiation of the primitive streak and impaired patterning within the anterior primitive streak (37). Other studies noted that Hhex expression lacked asymmetry in the anterior visceral endoderm of murine embryos and that TLE4 expression could also induce endodermal expression of Hhex (38, 39). Using a fluorescent marker to track Hhex expression in the early endoderm, the importance of Hhex for self-renewal was demonstrated with the absence of Hhex allowing cellular proliferation and differentiation (40). The requirement of Hhex in the endothelial tissues during murine embryological development of the forebrain was also reported, in addition to liver and thyroid, which may be related to Hhex polymorphisms as risk factors in neurological diseases such as Alzheimer’s disease discussed later in this review (41).

A link to β-catenin/Wnt signalling was first suggested when Hhex expression was ablated in the developing mouse embryo by conditional deletion of β-catenin at E7.5 in the prospective definitive endoderm of the neural plate stage embryos (**Figure 3**) (42). In addition, within the ventral foregut endoderm of the developing mouse embryo, SRY-Box Transcription Factor 13 (Sox13), a known Wnt/TCF signalling repressor, was shown to directly interact with Hhex, where Hhex blocks Sox13 repression of Wnt/TCF, whilst Wnt/TCF could in turn de-repress Hhex (**Figures 1**, **3**) (43). This implies the presence of a positive feedback loop in which Hhex can amplify Wnt/TCF signalling to drive development of the murine embryo.

The first reported target of Hhex in development was regulation of the sodium-bile acid cotransporter protein *via* a Hhex response element (HRE) in the promoter (44). It was also found that Hhex directly binds and represses endothelial cell-specific molecule-1 (ESM-1), *via* the evolutionarily conserved HRE-1, revealing the essential role of Hhex in the formation of the vascular endothelium in the developing embryo (45). Vasculogenesis and cardiac development was also found to require Hhex, with VEGFa levels repressed by Hhex in murine embryos (**Figure 3**) (46). It was also revealed in mice that urokinase-type plasminogen activator (uPA) induces angiogenesis *via* reducing the transcription activity of Hhex which causes de-repression of the VEGF receptor expression (47).

Liver and pancreatic development in the anterior definitive endoderm were also observed to be driven by Hhex^+^Cxcr4^+^ cells upon isolating and culturing the cells *in vitro (* 48). Hhex was also observed to induce liver development in an *in vivo* system, independently of Cxcr4 expression, with lack of Hhex also inhibiting pancreatic development (49). This study showed that Hhex controls the proliferation rate of the endodermal cells in the leading edge which allows it to grow beyond the cardiogenic mesoderm when the gut tube is closing and the positioning of these cells is essential for pancreatic specification (49).

Organogenesis in the murine embryo endoderm was demonstrated to be induced by Hhex by promoting hepatoblast development in the stromal environment by allowing continued differentiation (49, 50). Moreover, recent studies have shown that pluripotent stem cells expressing wildtype Hhex can facilitate normal liver development in both mice and pigs otherwise lacking Hhex, which in the absence of Hhex results in embryonic lethality (51).

Mechanistically, it was observed that HNF3β and GATA-4 motifs in the Hhex promoter transactivate Hhex in the liver allowing tissue-specific expression of Hhex (52). Using embryoid bodies, Hhex was shown to synergise with BMP-4, inducing upregulation of Albumin, Afp carbamoyl phosphate synthetase, transcription factor 1 and CCAAT/enhancer binding protein alpha, leading to secretion of Albumin and transferrin, and inducing a pro-hepatic gene signature which included fibrinogens, apolipoproteins and cytochromes (53). Hhex was also observed to repress Shh cell signalling in hepatocyte proliferation of the developing mouse embryo, by translocating to the nucleus and mediating transcriptional repression (54). This process was facilitated by GPC3 binding CD81, which would otherwise bind to Hhex, keeping it from entering the nucleus (54). The transcriptional repression by Hhex was also blocked *via* Shh, which itself is bound by GPC3, that in turn downregulates its function as well as it binding to CD81 (54). It was also reported using Hhex KO mice that Hhex is necessary for hepatic differentiation in the endoderm *via* VEGF signalling, independently of endothelial cells (55). An important function of Hhex in bile duct formation was also suggested using Notch2 KO mice, where Hhex expression declined perinatally, normalised post-weaning, and remained elevated in icteric 6-month-old mice, thereby suggesting a role in promoting secondary bile duct formation (56).

In a genome-wide computational analysis study, aimed at identifying cis-regulatory transcription factors, Hhex was reported to be controlling embryonic blood and endothelial development at E11.5 mouse embryos, shown *via* β-galactosidase reporters (57). The use of embryonic stem cell (ESC)-derived Hhex KO embryoid bodies revealed that they lacked macrophage potential whilst endothelial cells expanded (58). In contrast, Hhex overexpression in embryoid bodies reduces cell numbers by upregulating Flk-1 and increases the number of blast colony forming cells (BL-CFCs) formed with haemangioblast characteristics (58).

A potential role of Hhex within the bone of murine embryos was shown at E15.5, with changes to intracellular localisation of Hhex during development (59). The same group also observed Hhex expression in the chondrocyte cell line, ATDC5, which increased with differentiation, and when Hhex was overexpressed, it induced a necroptotic-like cell death (60). A role of Hhex in central nervous system (CNS) neurons during murine embryonic development was discovered by the observation of inhibition of axonal growth when Hhex is prematurely expressed (61). This may be linked to Hhex’s potential role in neurological diseases (see below).

### 2.3 Zebrafish

Zebrafish have been used to study the role of Hhex in the embryological development of vertebrates. This first study in zebrafish specifically observed Hhex expression with the yolk syncytial layer, equivalent to the murine visceral endoderm, acting as a transcriptional repressor (62). Initially, Hhex is regulated by the maternal Wnt pathway, and later by the Bmp-mediated pathway, with overexpression of Hhex downregulating both pathways, whilst concordantly upregulating chordin (62). Bmp and Fgf are required in liver development of zebrafish by specifically blocking Hhex and Prox1 expression within that tissue, but not in the neighbouring endoderm and mesoderm (63). In addition, within the dorsal yolk syncytial layer of zebrafish, Hhex is activated by Wnt/β-catenin, along with Vega1 and 2 *via* the action of Boz, which in turn allow the repression of Hhex (64).

Hhex is also vital for zebrafish haemangioblast development where it functions downstream of *Cloche*, a gene that plays an important role in haemangioblast differentiation (65). Scl and Hhex induce each other’s expression suggesting that they may also compensate for each other’s functions (65). A role for Hhex was also revealed as a transcriptional regulator of the VEGFC/FLT4/PRX1 signalling pathway that is necessary for development of the vascular system in zebrafish (66).

Normal hepatopancreatic duct (HPD) formation in the zebrafish embryo also requires Hhex, as shown using Hhex KO zebrafish (67). The need for Hhex in HPD formation was also verified in a subsequent study, where Hhex was shown to be necessary in both the endoderm and the yolk syncytial layer for HPD fate (68). The mutation of Telomere Maintenance 2 (Tel2) was observed to repress Hhex, whilst unmutated Tel2 engaged with Hhex’s promoter to facilitate Hhex expression necessary for normal liver regeneration in zebrafish (69).

Late thyroid development in zebrafish embryos was also observed to require Hhex, along with the homeobox transcription factor Nk2.1a, also known as Thyroid Transcription Factor-1 (TTF-1) in mammals (70). Indeed, another study also noted Hhex, along with Pax2a and Nk2.1, tightly regulated Bcl2l in the developing thyroid of zebrafish, implying a potential role of Hhex in regulation of pro-survival molecules (71), a finding that will be covered more extensively later in the context of lymphopoiesis.

### 2.4 Xenopus

Hhex was first identified in *Xenopus* by Newman et al, and denoted *Xhex*, where in the gastrula stage embryo, it was found to be expressed in the dorsal endomesoderm, which in turn gives rise to the liver (3). Hhex also plays an important role in anterior development originating from the endoderm (72) and was also observed to be important in vasculature development where it may have a role in the VEGF/Flk-1 signalling pathway in vascular endothelial cells (3). Overexpression of maternal Wnt/β-catenin and TGF-β signals induced ectopic Hhex and Cerberus, both early gene markers of the anterior endomesoderm, whereas blocking these pathways, downregulated expression of Hhex and Cerberus (73). Expression of the BMP antagonists Noggin and Chordin was found to allow normal Hhex and Cerberus expression, and conversely Hhex mRNA, injected ventrally, upregulated ectopic Cerberus (73). This research goes some way to describe the initial gene expression events associated with Hhex expression within the anterior endoderm required for normal development of the *Xenopus* foregut and liver (73). Another study suggested that Hhex promotes anterior identity in the *Xenopus* embryos by directly repressing Goosecoid, as well as being required for endodermal anterior patterning (9, 74, 75). These studies underscore the complex roles of Hhex in regulating the expression of multiple genes during the development of the *Xenopus* embryo.

Promoter analysis within the *Xenopus* embryos revealed that β-catenin represses Hhex expression indirectly *via* the homeodomain repressor Vent2, but conversely, subsequently drives liver organogenesis (76). Moreover, it was shown in both murine and *Xenopus* embryos that a 4.2kb upstream region of the Hhex gene was important for Hhex expression in endothelial precursor cells, liver and thyroid where an intronic component was required and adequate for normal anterior visceral endoderm and anterior definitive endoderm development (77).

Hhex is also required for normal cardiogenesis in *Xenopus* embryos, where its expression is induced in the endoderm *via* the Wnt/β-catenin signalling antagonist Dkk-1 and Hhex goes on to then regulate diffusible heart-inducing factor (78). Hhex, along with Cer1, was necessary for the Sox17 signalling pathway required for cardiac mesoderm formation in murine Embryonic Stem Cells (mESCs) (79).

Hhex plays a vital role in embryological development from organisms such as fish and amphibians, up to mice and humans. Universally, across all organisms analysed, Hhex is crucial for endodermal and mesodermal development within the embryo, giving rise to the foregut, thyroid, pancreas (in mice and humans), liver, haematopoietic and vascular systems. Hhex operates as a transcriptional repressor, in combination and in conflict with various other transcription factors and developmental proteins depending on the organism, but consistently in relation to Wnt/β-catenin signalling which appears to govern Hhex expression. Bmp is also consistently repressed by Hhex expression in mice, fish and amphibians, with the observation yet to be made in humans. Whilst in humans and mice, Shh is able to repress Hhex expression. These fundamental roles of Hhex in the formation of key organs and tissues in the developing embryo, foreshadow a continuing importance of Hhex in haematological and endocrinological diseases as discussed below.

## 3 Role of Hhex in HSC development and maintenance

As its name suggests, Hhex plays a central role in haematopoiesis in vertebrates with its haematopoietic expression detected across fish, amphibians, birds and mammals (1, 3, 62). The first haematopoietic progenitors found within the developing embryo are within the initial site of primitive haematopoiesis within the liver, the haemangioblast, and Hhex is essential for its development (80). Early studies revealed a high level of Hhex expression across many branches of haematopoiesis including within myeloid and erythro-megakaryocytic progenitor cells, with both lineages downregulating Hhex with differentiation, as well as within B cells, Natural Killer (NK) cells, dendritic cells (DCs) and immature T cell progenitors in the thymus (6, 7, 81). Whereas another study showed that, conversely, Hhex overexpression in haematopoietic progenitor cells results in a failure to contribute to mature blood lineages (82). An important role for Hhex was also demonstrated in erythropoiesis, specifically with regard to the globin genes, where Hhex is upregulated, along with Id2, in high-foetal haemoglobin conditions in human erythroblasts (83). Creation of murine Hhex KO ESC cocultures, where haematopoietic progenitor cells (HPCs) developed *in vitro*, showed that loss of Hhex delayed haemangioblast formation and caused an accumulation of CD41+ and CD41^+^/c-Kit^+^ cells, thought to be the earliest HPCs, as well as impairing further haematopoietic development by impeding their proliferation (84).

Our own studies revealed that Hhex was essential for murine HSC self-renewal and emergency haematopoiesis following myeloablation (85). In these settings, Hhex directly represses Cdkn2a *via* PRC2 complex-mediated repression, in a similar mechanism as observed in acute myeloid leukaemia (AML) (85, 86). Cdkn2a is highly upregulated when Hhex is deleted in both HSCs and AML, and the absence of Cdkn2a rescues the defective HSC self-renewal and emergency haematopoiesis observed in Hhex KO mice (**Figure 3**) (85, 86). The repression of Hhex, and resultant expression of Cdkn2a and Cdkn1b, was also noted to be necessary for osteoclastogenesis in mice, indicating similar relationship between Hhex and cyclin dependent kinase inhibitors in the context of osteoclasts (87).

Within both the embryo and adult, an evolutionarily conserved non-coding region in the Hhex locus was found to bind the important HSC transcription factors Gata2, Scl, Fli1, Pu.1 and Ets1/2 and to be essential for HSC development, haematopoiesis and homeostasis (88). The methyltransferase SETD8 was shown to be an erythroid specific repressor of Hhex, along with Gata2 and Hlx, with Hhex being upregulated when SETD8 was deleted (89).

These studies collectively illustrate the key role of Hhex in adult HSCs and haematopoiesis, continuing from Hhex’s necessity in the haemangioblast of the developing embryo. The repression of Cdkn2a *via* PRC2 by Hhex is central to its function and Hhex appears to utilise this mechanism in the context of AML. Being able to inhibit Hhex may therefore be a clinical strategy in the context of some haematological malignancies.

## 4 Role of Hhex in lymphopoiesis

Several laboratories have now shown that Hhex plays a pivotal role in lymphopoiesis. The impaired B cell development exhibited in Hhex null mice was first reported in the context of a RAG1-deficient blastocyst complementation system which circumvented the embryonic lethality of Hhex KO mice (90). This study noted deficiencies in mature B cells, pre-B cells and CD5^+^ B cells as well as the presence of a CD19^+^B220^-^ aberrant B cell population within the bone marrow of Hhex KO mice (90). Moreover, studies using Lck-Hhex transgenic mice showed that overexpression of Hhex in T cells impacted their development, demonstrating that downregulation of Hhex is necessary for normal T cell development (91).

A critical role for Hhex in early murine lymphopoiesis was most clearly demonstrated using haematopoietically inducible KO mice and competitive bone marrow transplantation assays, where the absence of Hhex blocked lymphoid cell development beyond the common lymphoid progenitor (CLP) cell stage (81). This defect in lymphopoiesis was characterised by the formation of a Pro-B-like aberrant (CD19^+^B220^-^) B cell population which was defective in IL-7/Stat5 signalling capacity with an increased level of apoptosis in the few remaining B cell progenitors (81, 92). However, expression of constitutively active Stat5 transgene failed to rescue the defective lymphopoiesis observed in the absence of Hhex, indicating that defective IL-7 signalling in this context was not the primary cause of the lymphopoietic defect observed (93). In contrast, transgenic expression of the potent anti-apoptotic molecule, Bcl2 was able to restore normal lymphopoiesis in Hhex null mice, both *in vitro* and *in vivo*, thus showing that Hhex plays a key role in inhibiting apoptosis during lymphopoiesis (**Figure 3**) (93).

Hhex-null CLPs exhibited downregulation of the cell cycle gene, Cyclin D1, which was shown to play a key role in the lymphoid developmental block, as retroviral overexpression of Cyclin D1 rescued lymphopoiesis *in vitro* (**Figure 3**) (81). Interestingly, in the human myeloid cell line (U937), Hhex was a reported as a negative regulator during translation of eIF4E protein, which in turn inhibited eIF4E-dependent transport of Cyclin D1 mRNA within the cell (23, 94). It was also demonstrated by the same laboratory that loss of Hhex resulted in aberrant nuclear function of eIF4E, where eIF4E is normally required for nuclear transport of Cyclin D1 mRNA into the cytoplasm (23, 94), a process stimulated by HOXA9 (24). Additionally, whilst the crucial cell cycle inhibitor Cdkn2a was observed to be upregulated in the absence of Hhex, its absence did not restore the impaired lymphopoiesis observed in the Hhex null mice, thus collectively suggesting that regulation of cell cycle is not the primary role of Hhex in the context of lymphoid development (93).

Beyond lymphoid development, Hhex plays specific downstream roles in both T regulatory cells (Tregs) and NK cells. The expression and function of Foxp3, the critical transcription factor required by Tregs, is negatively regulated by Hhex, which binds directly to the Foxp3 locus, with Hhex overexpression resulting in a failure to suppress the immune response in murine models of Treg function (95). In contrast, TGF-β/Smad3 signalling, which promotes Treg activity, was found to downregulate normal Hhex expression in Tregs (95). In the context of NK cells, repression of Hhex expression is required for normal development (96). Conversely, Hhex was shown to directly repress the expression of the pro-apoptotic protein BIM to allow normal NK cell survival (97).

Recently, Hhex, in concert with transcriptional corepressor TLE3, was also revealed to be a key regulator of germinal centre B cells developing into memory B cells through induction of the transcription factor Ski (98). The absence of Hhex in memory B cells resulted in an upregulation of Bcl-6, which was also shown to directly repress Hhex in germinal centre B cells (98). Similar to its function in CLPs, the Bcl-6 target gene, Bcl2 was able to rescue the Hhex KO phenotype in memory B cells (**Figure 3**) (98). This suggests that the significant role of Hhex in maintenance of cell survival remains important throughout B cell development.

## 5 Role of Hhex in leukaemia

Given the vitally important role of Hhex in haematopoiesis, it is no surprise that Hhex has increasingly revealed itself as playing a fundamental role in both the development and maintenance of various haematological malignancies, most notably in the context of T-ALL and AML. Upregulation of Hhex expression was first seen in the peripheral blood of B cell leukaemia patients (99) and dysregulation of Hhex was then subsequently suggested to be a contributing factor to B cell leukaemogenesis (7). Hhex was found to induce murine B cell leukaemia in the AKXD model as a consequence of retroviral insertion upstream of Hhex and mEg5 (100). In this system, both Hhex and mEg5 were upregulated following retroviral insertion but only Hhex was expressed highly in these samples (100). Subsequent studies using bone marrow transplants in lethally irradiated recipient mice of retrovirally overexpressed Hhex in HPCs showed Hhex induced T cell lymphomas (82).

Whilst these findings indicate the potential involvement of Hhex in B and T-cell leukaemogenesis, in the case of lymphoma one group noted a clear reduction in Hhex expression in all human B cell lymphoma classes they tested, with the exception of oncogenic activation (101). Indeed, in some primary cutaneous T cell lymphoma (CTCL) patient samples HHEX was shown to be deleted (102). Specifically, the deletion of HHEX *via* a 10q23.33-10q24.1 chromosomal deletion resulted in a loss of heterozygosity in about half of the patient samples, thereby being suggestive of a role for HHEX in the aetiology of CTCL (102). Although, in the context of anaplastic large cell lymphoma (ALCL), HHEX was not observed to drive the pathogenesis of disease, as its overexpression induced apoptosis and differentiation and its expression was repressed by TGFβ/SMAD-pathway in ALCL cell lines (103).

The utility of Hhex has also been strongly established in the development of T-ALL in both mice and humans. The clearest evidence for this was shown in a murine model Lmo2-induced leukaemia in mice and T-ALL patient samples, where it was revealed upregulation of Hhex as an integral part of a broader induction of an HSC transcriptional programme and where Hhex could additionally phenocopy the action of Lmo2 in early leukaemogenesis (**Figure 3**) (104). In a study using Rag-deficient NOD mice, T-ALL initiation was potentially caused by the loss of T cell progenitor checkpoint regulation, with induction of a HSC gene programme including Hhex, as well as Lmo2, Lyl and Kit (105). Indeed, the +1 enhancer element of HHEX was shown to be directly bound by LMO2/FLI1/ERG in human T-ALL (14). HHEX was also observed as a direct transcriptional target of LMO2 in human early T-cell Precursor (ETP)-ALL (106). The same group also found CD2-Lmo2 transgenic mice required Hhex to be expressed for development of T-ALL, implying Hhex as a crucial mediator of the oncogenic functions of Lmo2 (106). We also observed that Hhex is required for the radio-resistance of Leukemic Stem Cells (LSCs) in a similar mouse model of human ETP-ALL (107).

Deacetylation treatment was also observed to downregulate Lmo2 expression and its target Hhex in T-ALL (108). Ldb1 and Lmo2 were also reported to bind the promoters of Hhex, Lyl1 and Nfe2, resulting in their upregulation in HSPCs and human ETP-ALL cell lines, as well as pre-leukaemic Lmo2 transgenic thymocytes in the murine Lmo2-induced T-ALL model (109). Induced deletion of Ldb1 conversely downregulates Hhex expression in murine T-ALL (109). Hhex was observed to be repressed by NKK-3 in human T-ALL samples. The same group noted that HHEX activated AUTS2, part of the chromatin modulating PRC1 complex, which in turn mediated MSX1 expression (110). Collectively, these studies underscore the interplay between other transcription factors, especially Lmo2 and its binding partners Ldb1 and Lyl1, in regulating the expression of Hhex leading to the development of T-ALL.

Dysregulation of Hhex is also well-documented in terms of its involvement in AML where nuclear Hhex was downregulated, whilst eIF4E was upregulated (23). Use of CD11c-Hhex transgenic mice revealed that high levels of Hhex during myeloid development may induce myeloid leukaemia, with higher cell cycle rates observed, although leukemogenesis was slow (18 months of age), implying that further mutation(s) were required in addition to Hhex overexpression (91).

Another group discovered an AML patient with a NUP98/HHEX chromosomal translocation as the only cytogenetic aberration and made a murine version of this genetic lesion (111). With a 9-month latency, the bone marrow bearing this lesion gave rise to a transplantable acute leukaemia, bearing similar gene dysregulation found in the more clinically common homeobox gene fusion NUP98/HOXA9 translocation (111). AMLs driven by NUP98-Hhex fusion, along with other NUP98-oncoprotien fusions, exhibit an induced aneuploidy *via* a weakening in the mitotic spindle checkpoint (112). Indeed, in the most commonly observed form of numeric aneuploidy in AML, trisomy 8, the HHEX gene body is repressed by hypermethylation and may serve as a potential diagnostic feature of the disease (113). Hhex overexpression was also detected in AML patients with the t (8, 21)(q22;q22) translocation and studies in Kasumi-1 cells, a leukaemic cell line which bears the 8:21 chromosomal translocation, showed that Hhex was required for their survival (114). These observations point to Hhex, and other Hox genes, as being both gene fusion partners and drivers for the promotion of AML development.

In the K562 myelogenous cell line, Hhex was shown to influence leukemogenesis through repression of VEGF *via* its promoter region, but required TLE co-repression to mediate its function (115). Dasatinib, a BCR-ABL/Src kinase inhibitor, reduced phosphorylation of Hhex, which in turn allowed Hhex-mediated repression of VEGF and VEGFR-1 leading to a reduction in cell survival (116). In the context of Acute Pro-myelocytic Leukaemia (APL) analysis of 18 patients showed PML-RAR-α reduced HHEX expression by targeting its promoter, which then downregulated VEGF-A, and thus the pro-angiogenic response in APL (117).

We have shown using a murine model of AML, specifically MLL-ENL, that Hhex was required for both the initiation and propagation of AML, with loss of Hhex resulting in the upregulation of p16^INK4a^ and p19^Arf^, leading to myeloid differentiation and growth arrest (86). Mechanistically, we demonstrated that Hhex represses PRC2-mediated epigenetic repression of Cdkn2a by binding to the Cdkn2a locus and directly interacting with the PRC2 to enable H3K27me3-mediated epigenetic repression (86). Hhex was also observed to be a direct target of Runx1, a transcription factor with known tumour-suppressor function, where Hhex combined with Flt3-ITD to induce AML in mice (**Figure 3**) (118). Hhex expression, in combination with a mutant form of additional sex combs-like 1 (Asxl1) an epigenetic modulator often mutated in myeloid leukaemia, was also found to enhance Runx1-ETO and Flt3-ITD-driven myeloid leukaemia *via* upregulation of Myb and Etv5 in mice (119). We also observed that Hhex overexpression induced self-renewal of murine IL-3 dependent promyelocytes *in vitro (* 120). Moreover, this function of Hhex required nuclear localisation and structure function analysis demonstrated a requirement of the DNA-binding and N-terminal–repressive domains of Hhex for promyelocytic transformation (120). Despite Hhex containing a PML-interaction domain (**Figure 1**), it did not require PML for transformation, nor did it require p16^INK4a^ and p19^Arf^ indicating Hhex did not require PRC2-mediated epigenetic repression for this particular process unlike what we observed for the induction of AML (120). Nevertheless, Hhex could still cooperate with growth factor (IL-3) independence to cause pro-myelocytic leukaemia in mice (120). It is increasingly clear that Hhex plays a vital, but context dependent, role in the pathology of AML, but typically requires cooperative mutations in growth factor signalling pathways.

In summary, across multiple types of haematological malignancy, Hhex appears to be a key player in the disease development. Whilst technically challenging, greater focus should be placed on developing effective ways to target Hhex and its interacting partners in leukaemia patients. There would be clinical benefit in being able to effectively drug Hhex, and related transcription factors, particularly in the context of AML, where in many countries an aging population is resulting in a dramatically increasing disease burden of AML, and where existing therapies are currently limited and suboptimal.

## 6 Role of Hhex in solid cancers

In addition to Hhex’s well-established roles in leukaemogenesis, it has been reported to contribute to the development of a range of solid tumours including those with endocrine functions such as in the breast, prostate and thyroid, as well as the liver, cervical and bile duct cancers.

Several studies point to Hhex playing an important part in the development of breast cancer. Hhex is expressed in breast epithelial cells, with its intracellular localisation regulated and altered by malignancy of these cells (121). Hhex was also noted to upregulate the NIS (sodium iodine symporter) promoter which is specifically upregulated in breast tissue with lactation (121). Work using a breast cancer cell line (MCF-7) showed that Hhex transcriptionally controlled endoglin and inhibited cell migration (122). Subsequent work from the same laboratory reported that siRNA Knock Down (KD) of Hhex in breast cancer cells enhanced their proliferation in part due to VEGF signalling (**Figure 3**) (19, 123). Moreover, Hhex overexpression impaired breast tumour growth in mice, which may help explain the poor prognosis which is associated with breast cancer patients exhibiting low Hhex expression (123). HHEX expression was also confirmed to be lower in human breast cancer compared to pre-cancerous tissue, potentially contributing to the worse clinical outcomes observed in breast cancer patients bearing low levels of HHEX expression (124, 125). In addition, type 2 diabetes (T2D) single nucleotide polymorphisms (SNPs) in Hhex (rs11187146) and Cdkn2a/b (rs1333049) were linked as being as additive risk factors in likelihood of developing and dying from breast cancer in an American patient cohort (126). Overall, these studies suggest that lower Hhex expression is a poor prognostic indicator in breast cancer and further study is needed to better understand its function in this disease.

In prostate cancer, the protein kinase CK2 was shown to impede Hhex by phosphorylation-induced inhibition of Hhex’s DNA binding, allowing increased proliferation and migration of prostate cancer cell lines (127). In addition, inhibition of CK2 blocked Hhex phosphorylation resulting in reduced cell proliferation (127). The same laboratory previously suggested that Hhex controlled the expression of endoglin in the inhibition of prostate cancer cell line migration (123). In the prostate cancer cell line PNT2-C2, TGF-β signalling downregulated Hhex expression, whilst also increasing Hhex phosphorylation (**Figure 3**) (128). Additionally, when looking at another endocrine organ, the thyroid, and given the vital role Hhex plays in its development, it was perhaps unsurprising to find Hhex reported as highly expressed in thyroid patient tumour samples with nuclear localisation (129).

It may be expected, given its important role in development of the liver, that Hhex may play a role in cancer development and progression within this organ. Indeed, the absence of Hhex appears to be necessary for the progression of hepatocellular carcinoma (HCC) with Hhex overexpression increasing known tumour suppressor genes p53 and Rb, whilst downregulating c-Jun and Bcl2, well known proto-oncogenes (130). These observations also correlated with reduced tumorigenicity in mice, with Hhex expression denoting poorly differentiated HCC, suggesting that absence of Hhex expression may serve as a biomarker of HCC progression (130). Studies of HCC have also revealed that Hhex interacts with the potent oncogenic transcription factor, c-Myc (131). KD of Hhex using siRNA showed increased proliferation in HCC (131). Whilst c-myc drives metabolism and proliferation, Hhex appears do the opposite, causing decreased c-Myc activity and reduced tumour growth in a murine xenograft model of HCC (131). However, another study found that Hhex was nevertheless expressed in the majority of HCC cell lines (132).

In cholangiocarcinoma (CCA), more commonly known as bile duct cancer, Hhex was also found to be highly expressed and to operate in a positive feedback loop with Notch3, which itself is important in CCA, as well as inducing Wnt signalling (133). CCA tumour growth was reduced with siRNA KD of Hhex in a xenograft model, and Hhex overexpression in cholangiocytes increased their proliferation (133). Interestingly, whilst Hhex is suggested to be a positive regulator in the context of bile duct cancer, in contrast it appears to operate as a negative regulator in the context of liver carcinoma, which may hint at the underlying of role of Hhex in embryological development of these two organs.

The importance of the methylation status of the Hhex gene was noted in melanoma patients, where those with hypermethylated Hhex exhibited significantly worse levels of overall disease-free survival, as well as disease specific survival and lymph node metastasis, compared to patients with hypomethylated Hhex gene (134). The methylation status of Hhex was also shown to be relevant in cervical squamous cell carcinoma (CSCC), where hypomethylated HHEX was also observed as a positive prognostic indicator in patients (135). Moreover, another study uncovered HHEX as a potential biomarker in CSCC, speaking to its importance in the pathology of the disease (136).

In summary, in addition to Hhex’s well-established role in haematological malignancy, the absence of Hhex, and in some settings its overexpression, serves as important drivers of solid tumour development, potentially stemming from its role in the embryological development of the organs from which the cancer is derived. These observations suggest that a better understanding of how Hhex mediates its normal developmental as well as its aberrant tumour-promoting functions may aid the development of more targeted therapeutics for cancer patients.

## 7 Role of Hhex in pancreas and diabetes

As previously discussed, Hhex plays a vital role in the embryological development of the organs of the vertebrate foregut including the pancreas. Moreover, Hhex also remains functionally relevant in the pancreas in the adult organism. Specifically, it was revealed within adult pancreas that Hhex is expressed in somatostatin-secreting delta cells (137). Use of two mouse models of pancreatic deletion of Hhex showed it is needed for pancreatic development (137). Moreover, decreased somatostatin in Hhex KO pancreatic islets caused impaired paracrine inhibition of insulin released from beta cells (137). In beta cells the Hhex locus is targeted by Lsd1 which facilitates H3K3me1/2 methylation-mediated repression of Hhex preventing beta to delta cell transition (**Figure 3**) (138). This suggested that compromised paracrine control may be partly responsible for T2D through the acceleration of beta cell exhaustion and failure (137). Hhex RNA and protein was also revealed in humans as being highly expressed in the pancreas, specifically the islets, exocrine acini and ductal epithelium, but not detected at significant levels in liver parenchyma and colonic epithelium (139). The overexpression or KD of Hhex in *Xenopus* showed that it is essential for the ventral pancreas formation, *via* Vpp1 expression in ventral pancreatic progenitor cells, as well as liver development (140). This finding was also verified in *Drosophila* in that Hhex is equally important in glucose metabolism, as revealed in tissue specific KD studies (141).

Increasingly, and perhaps unsurprisingly, Hhex has been observed as a notable risk factor in a number of endocrinological and metabolic diseases that involve the pancreas. A number of allelic SNPs (rs1111875, rs5015480 and rs7923837) within the Hhex gene have been implicated to varying degrees as T2D risk factors, with a Genome-Wide Association Study (GWAS) linking rs5015480 with gestational diabetes mellitus with these studies and meta-analyses showing that the ethnic background of the patient population is the most important factor as to whether a Hhex SNP risk factor allele applies and to what extent (**Supplementary Table 1**) (142). Indeed, a study of T2D patients using ATAC-seq also detected Hhex in open chromatin peaks, amongst other candidate genes associated with T2D and islet dysfunction (143). In murine studies, Hhex may potentially play a broader role in metabolism beyond the pancreas, such as in the liver which was shown to have high Hhex expression, but which decreased in response to high fat feeding (144). This study however also conflicts with that of Costapas et al, who reported that pancreatic islets exhibited lower Hhex expression (139, 144). Nevertheless, the modulation of Hhex expression within the liver in response to dietary metabolism suggests that Hhex SNPs may play a relevant role as a risk factor in T2D susceptibility (144).

Interestingly, Hhex may function in the pancreas *via* a similar fashion to that which was observed in HSCs and leukaemia, by directly repressing Cdkn2a, as a consistent SNP in the Cdkn2a gene (rs10811661) is often concurrent with SNPs within the Hhex gene as well-established risk factors in the development of T2D (**Supplementary Table 1**). Indeed, in a study of T2D patients Hhex and Cdkn2a polymorphisms were detected in about half of patients, where it was shown a CpG site was introduced or removed associated with the differential methylation the SNP-CpG site of Hhex in pancreatic islets (**Figure 3**) (145). Moreover, Cdkn2a, along with several other genes, was also associated with both differential methylation of DNA of the CpG-SNP site within islets and the DNA methylation of surrounding CpG sites, suggesting that this may be a molecular means by which Hhex SNPs associated with T2D mediate their effect in patients (145).

There were also conflicting separate studies regarding the role of T2D SNPs affecting low birth weight when inherited by the offspring (**Supplementary Table 1**). Given the role of Hhex SNPs in T2D and glucose metabolism, its influence on the risk of T1D development, polycystic ovary syndrome (which shares an insulin resistance link with T2D) and metabolic syndrome was also explored in humans with studies revealing no such link from several GWAS studies (**Supplementary Table 1**). Interesting though, Hhex’s association with T2D, which extends to high body weight index, may also have further a role in adipocyte development *in vitro* where lack of Hhex impairs expression of PPAR-gamma protein and impedes adipogenesis (146). Based on the evidence produced thus far, Hhex does not appear to play a role in birth weight, PCOS, metabolic syndrome or T1D development despite a clear relationship with glucose metabolism in T2D, but it may be involved in adipocyte development.

Several studies have hinted at how Hhex may be regulated and which functions it performs within the pancreas. Hhex is upregulated in human islets by gastrin hormone treatment (50) and Aldh1a2 KD reduced Hhex expression, along with Prox1, in the pancreas and liver (147). Using hESCs it was shown that Hhex, along with Pax6, may be repressed by Aristaless related homeobox (ARX) in that ARX KO pancreatic progenitor cells exhibited an upregulation of Hhex and conversely when ARX was re-expressed, Hhex was then downregulated (148). Whilst Hhex is not required in ductal cell function of adults, KD of Hhex in pancreatic progenitor cells can cause pancreatitis (149). However, Hhex is vital in early life for maintenance of ductal homeostasis and allowing ductal hypersecretion as a cause of chronic pancreatitis in children (149). Ferreira et al. went on to show that the G-protein coupled receptor Npr3 is repressed by Hhex and thereby the potential secretion by ductal cells (149). Within islets, delta cell specific-Hhex was shown to control cAMP and concentration of intracellular calcium *via* histone post-translation changes, which in turn modulates Cav1.2 calcium channel and adenylyl cyclase 6 (AC6) and secretion of somatostatin (150). These histone modifications that epigenetically control secretion of somatostatin within islets were mediated by a super complex composed of the Cullin 4B-RING E3 ligase (CRL4B) and interestingly, the PRC2 methyltransferase complex (**Figure 3**) (150).

The strong association of SNPs risk factors in T2D for both Hhex and Cdkn2a across a broad spectrum of human ethnicities, combined with observations that epigenetic modifications made *via* Hhex within pancreatic islets involve PRC2, are tantalising. This strongly suggests that Hhex’s well-documented function in HSCs and leukaemia *via* PRC2-mediated repression of Cdkn2a may also be one of its primary roles in the adult pancreas. However, further research is still required to resolve this hypothesis more conclusively.

## 8 Role of Hhex in endocrinology

Perhaps unsurprisingly given its important role in the developing thyroid gland and pancreas in the embryo, Hhex continues to play an important part in the endocrine system and in endocrinological diseases. The expression of Hhex was observed in both early undifferentiated thyroid cells and in the adult thyroid gland of both rats and humans, as well as in differentiated follicular thyroid cell lines (151, 152). Cells of the thyroid line FRTL-5 decreased their levels of Hhex expression in response to thyroid stimulating hormone (TSH) (152), with another study in differentiated human thyroid cells reporting that Hhex was not required for thyroid-specific gene expression induced by TSH (153). The thyroglobulin promoter was shown to be repressed by Hhex, which in turn blocked the activation of thyroid transcription factor-1 (TTF-1, also known as NKX2-1) and Paired box 8 (Pax8) (**Figure 3**) (152). In a subsequent publication, the same laboratory noted that TTF-1 enhanced the promoter activity of Hhex in rat FRTL-5 cells, and that the mRNA of both TTF-1 and Hhex was co-expressed in human thyroid tissues (154). In another report following on from that work, Puppin, et al, identified a relationship between Pax8 and Hhex, where Pax8 induced Hhex protein expression in a thyroid cell line and induced Hhex promoter activity in non-thyroidal cell lines (155). Hhex, along with Pax8, Foxe1 (Forkhead Box E1, also known as TTF-2) and E-Cadherin, were also observed to be downregulated in response to the functional inactivation of TTF-1 in PCCI3 thyroid cells (156). Whilst Hhex has minimal impact on thyroid specific gene expression (153), Foxe1 is required for NIS expression as shown in FRTL-5 cells (157). And it is also worth noting that Hhex upregulates the NIS promoter within breast tissue (121), suggesting a potentially similar mechanism of function for Hhex within both the breast and thyroid. Indeed, in the precursor cells of developing thyroid Hhex, along with Pax8, TTF-1 and Foxe1, operate in a highly inter-related network governing normal thyroid development (**Figure 3**) (35, 158, 159). Collectively, this research clearly shows the close relationship between the transcription factors Hhex and Pax8 in regulating TFF-1 expression to govern thyroid function.

Potentially conflicting reports exist showing that Hhex was absent in oncogene-transformed thyroid cell lines (Pellizzari, 2000), however another study from the same laboratory showed that Hhex was actually highly expressed in thyroid tumour samples from patients and concentrated within the nucleus (129, 152). Hhex, along with notably Pax8 and NIS amongst other thyroid specific genes, was also observed to be significantly decreased in patients in both benign thyroid tissues and carcinomas suggesting a potential involvement in a de-differentiation process (160).

Given the well-established role of Hhex in thyroid development within the embryo, the effect of mutations within Hhex was examined. Although Hhex mutations were found to not be a driver of thyroid dysgenesis (TD), PAX8 R52P mutation was implicated (161). Whilst another group subsequently examined the thyroid tissue of Chinese children suffering from TD for Hhex mutations (162), they also failed to show any link between Hhex mutations and TD, along with FOXE1, TTF-1 and PAX8, but still observed a correlation with the intronic mutation rs2275729, although owing largely to the small study size, further work is required to determine its potential importance (162). In addition, heterozygous Hhex mutations were detected in a small fraction (8/110) of congenital hypothyroidism patients which ultimately went on to develop TD (163).

The adrenal gland may also have a requirement for Hhex, with a meta-analysis of patients detecting a Hhex SNP (rs2497306) associated with levels of serum dehydroepiandrosterone sulphate (DHEAS), which is produced by the adrenal gland and associated with aging (164). Moreover, the rs2497306 SNP was also observed to be negatively associated with serum DHEAS levels of female RA patients (165). Additionally, the mild endocrine disruptor DDT (Dichloro-diphenyl-trichloroethane) was found to disrupt the Hhex-mediated regulation of cellular proliferation within rat adrenal cortex (166). These observations collectively suggest a role for Hhex in regulating processes within the thyroid and adrenal glands that warrant further investigation.

## 9 Miscellaneous roles of Hhex in neurological and other diseases

A number of studies have examined the potential role of Hhex in Alzheimer’s Disease (AD) in terms of SNPs that are known to be risk factors in T2D. However, several GWASs ultimately concluded that Hhex SNP rs1544210 was not specifically associated with late-onset AD (167–169). However, another meta-analysis found whilst Hhex SNP rs1544210 was not statistically significant in analysis of their 3 included studies (p=0.04, 0.09 and 0.29), there was a trend towards association with late-onset AD susceptibility (170). In a European patient cohort study of 110 candidate polymorphisms, Hhex SNP rs1111875, a major risk factor in T2D, was found to be a highly significant risk factor (p<0.00001) for AD, but only with the accompanying GSTM3 (rs7483) SNP (171). Whereas in a Korean population it was shown that Hhex polymorphisms observed in T2D (rs1111875 and rs5015480) were not associated with AD or Parkinson’s Disease (PD) (172), Hhex T2D SNP rs1544210 was associated with greater dementia and AD risk in a Swedish population (173).

Simpson et al. investigated how Hhex may influence neuronal biology, noting that Hhex had broad expression in CNS neurons in adults, including neurons of the corticospinal tract following spinal damage, and was amongst the most potent inhibitors of neurite growth (61). However, in adults Hhex expression was substantially reduced in immature cortical and peripheral neurons (61). In early immature cortical neurons, Hhex overexpression impaired both the initial axonogenesis including the axonal elongation growth rate with domain deletion analysis suggesting Hhex acted in this context as a transcriptional repressor (61). In the context of multiple sclerosis (MS), the HHEX SNP rs7923837, is a known risk factor of the disease (174). This observation may be related to more metabolically active lymphocytes in the blood of MS patients, which also express significantly less HHEX, but also bear far greater nuclear rs7923837 SNP Hhex, when compared to healthy controls (174). Recently, the microglia of mice were shown to decrease their Hhex expression when socially stressed or administered with agonists to TLR-2 and TLR-4 (175). Conversely, Hhex overexpression dampened the expression of inflammatory genes associated with TLR-4 induction, collectively suggesting that Hhex may be repressed by inflammatory signals (TLR-2/4) which can then contribute to neuro-inflammation in microglia (175). These findings suggest the potential of therapeutic intervention targeting Hhex in the treatment of neuro-inflammation in certain disease settings.

There are also a number of reports of Hhex function in various aspects of physiology including angiogenesis, milk production, muscle and lung function, as well as various diseases including psoriasis, hepatic and gallstone disease. In a study of milk production in dairy cows Hhex was reported to be targeted by miR-148 and regulate VEGFA, NRP1 and MYH10 with these genes in turn targeted by miRNAs miR-186, miR-148 and miR-141/200a respectively (176). A potential role for Hhex in terms of lactation, specifically protein localisation, was noted *in vitro* and Hhex may also play a role in mammary cell differentiation and tumorigenesis (121). In lung fibroblasts, Hhex expression was induced in response to TGF-β1, as was miR-21-3p which targets Hhex (177).

In muscle, Hhex was observed to upregulate gene expression of SMemb/Non-muscle Myosin Heavy Chain-B *via* the cAMP-Responsive element (178). In vascular smooth muscle cells, Hhex overexpression promoted G0/G1 to S-phase cell cycle transition, inducing cell cycle genes including CDK2, CDK6, CyclinB2 and CyclinD2, and inhibiting apoptosis, with the authors linking this to a potential role in vascular proliferative disease (179). Indeed, another study noted Hhex promoted vasculogenesis *via* VEGF as it was associated with increased vascular density in a rat model of stroke (180). In the skin lesions of psoriasis patients, Hhex mRNA and protein was found to be significantly lower in mesenchymal stem cells which suggested a role for Hhex in angiogenesis *via* its known influence on the VEGF signalling pathway (181, 182).

Hhex was also shown to be a novel bile acid-induced FXR/Fxr target gene following chronic bile acid exposure in hepatocytes with the FXR/Fxr binding to a conserved intronic enhancer in both human and mouse Hhex (183). The prevalent Hhex T2D risk factor SNP rs1111875 was found to be significantly associated with development of gallstone disease and is suggested as a potential biomarker (184). Additionally, Hhex was shown to be necessary in the formation of hepatic cysts of the bile duct in a liver conditional KO model in mice, resulting in increased expression of PC1/2 in the absence of Hhex (185). These data suggest Hhex may play an important role in various liver diseases.

**Figure 4:**
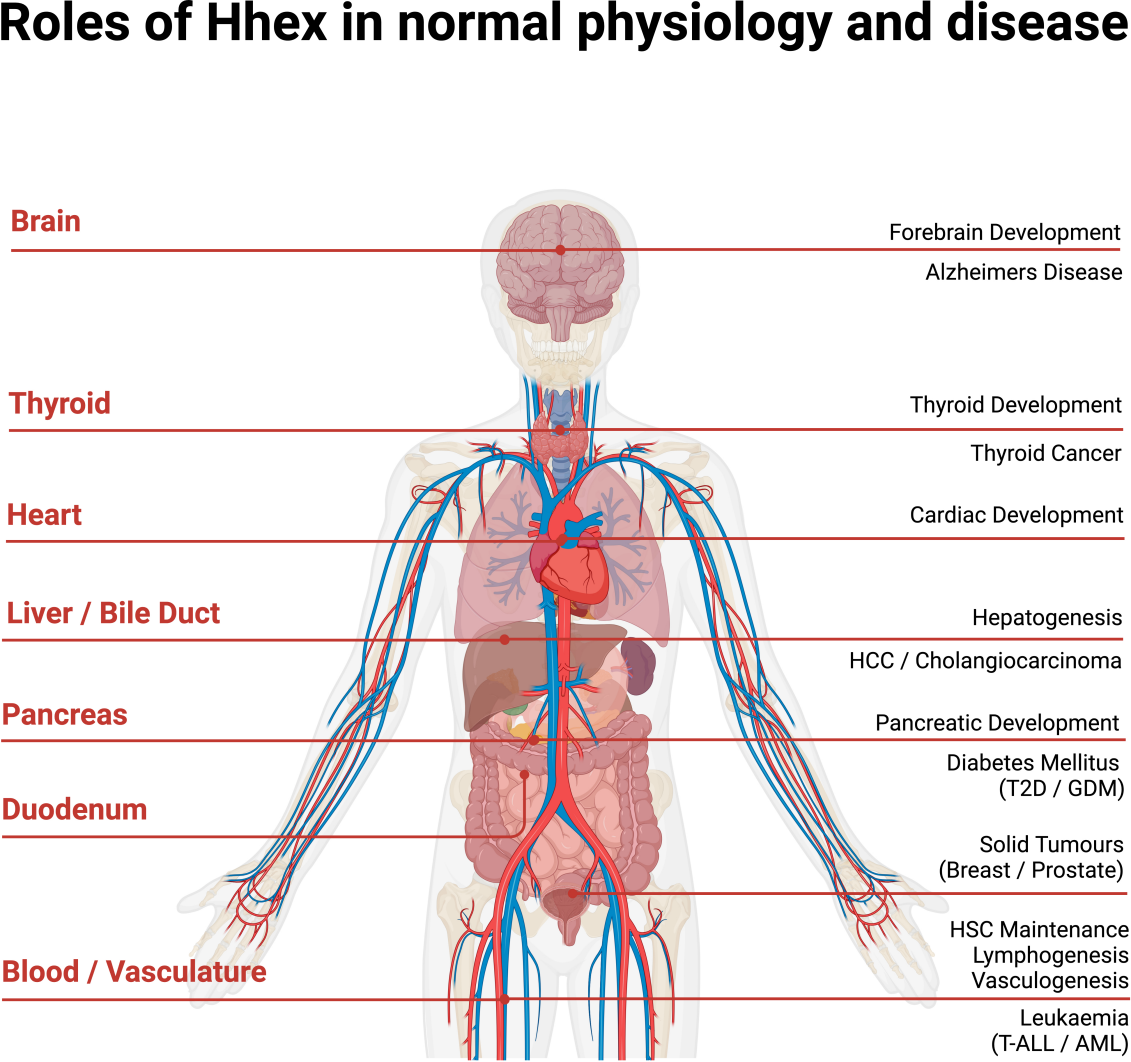
Anatomical overview of Hhex in normal human physiology and disease. Acute Myeloid Leukaemia (AML), Gestational Diabetes Mellitus (GDM), Haematopoietic Stem Cell (HSC), Hepato-Cellular Carcinoma (HCC), Type II Diabetes (T2D), T cell Acute Lymphoblastic Leukaemia (T-ALL). Created with BioRender.com.

These seemingly disparate involvements of Hhex in various organs and tissues all likely hint at a continuation of the utility of Hhex beyond embryonic development (**Figure 4**). For example, Hhex was noted as playing an important role in the development of liver, vascular endothelium and forebrain which may link to the reported observations above. Hhex may well also have undocumented functions in breast, muscle and myelination during embryology. Further research into the Hhex’s functions in both development of the embryo and adult will elucidate more clearly if its functions are maintained in the adult or whether it is redeployed in additional roles.

## 10 Conclusions/perspectives

This review reveals that Hhex is a crucial transcription factor throughout vertebrate evolution and the lifespan of the organism from embryo to adult. Hhex acts as a powerful transcriptional repressor, notably of PRC2 target genes such as Cdkn2a in HSCs, leukaemia and potentially in diabetes, given that SNPs in Hhex are typically noted as a risk factor alongside Cdkn2a. Hhex also plays a distinct role in maintaining pro-survival genes during lymphopoiesis. Additionally, Hhex itself appears to be regulated during embryological development by the Wnt/β-catenin signalling pathway in which it operates in a positive feedback loop. Moreover, Hhex is reported to repress genes in many other contexts including Eomes in hepatogenesis, Sox13 in the foregut endoderm, ESM-1 in the vascular endothelium, VEGF in vasculogenesis and cardiogeneisis, goosecoid in *Xenopus* anterior identity and the thyroglobulin promoter governing TTF-1 and Pax8 in the thyroid gland (**Figure 3**). Many of the diseases where Hhex manifests as a driving or contributing factor echo Hhex’s embryonic functions within the affected organ, where Hhex continues to play an important role. Thus, more extensive research into the exact role of Hhex in haematological malignancies, solid tumours, diabetes and thyroid diseases, may offer the greatest immediate benefits for diseases where Hhex is already heavily implicated and greater therapeutic intervention is still required. As such more broadly, further study into Hhex’s precise mechanisms of action and direct binding partners may contribute to tackling disruptions to embryonic development, diseases of the adult endocrine system and malignancies.

## Author contributions

JJ wrote the manuscript and created the figures and table. MM helped plan the manuscript. MM and SN provided invaluable feedback in drafting the manuscript, figures and table. All authors contributed to the article and approved the submitted version.
