# Supplementary material for: The Haematopoietically-expressed homeobox transcription factor: roles in development, physiology and disease
Source: Front Immunol. 2023 Jun 16;14:1197490. doi: 10.3389/fimmu.2023.1197490 (PMC10313424; doi:10.3389/fimmu.2023.1197490)
Supplement: Supplementary file 1 [file Table_1.docx]

**Table 1. Hhex Single Nucleotide Polymorphisms and Disease Association Studies**

| ***Hhex SNP Allele(s)*** | ***Cdkn2a/b SNP Allele(s)*** | ***Patient Population*** | ***Disease*** | ***Study Type*** | ***Other Findings*** | ***Reference*** |
| --- | --- | --- | --- | --- | --- | --- |
| rs1111875, rs5015480 and rs7923837 | rs10811661 | Korean | GDM | GWAS |  | *[*[*1*](#_ENREF_1)*]* |
| *rs5015480* | NR | *Swedish* | *GDM* | *GWAS* | *Not a risk factor for GDM* | *[*[*2*](#_ENREF_2)*]* |
| rs5015480 | rs10811661 | Swedish | GDM | GWAS |  | *[*[*3*](#_ENREF_3)*]* |
| rs5015480 | NR | Polish | GDM | GWAS |  | *[*[*4*](#_ENREF_4)*]* |
| rs5015480 | NR |  | GDM | Meta-analysis | (From 4 studies) | *[*[*5*](#_ENREF_5)*]* |
| Unable to source paper | Unable to source paper | Korean | GDM - early, postpartum pregnancy | GWAS | T2D SNPs associated with early post-partum GDM pregnancy | *[*[*6*](#_ENREF_6)*]* |
| rs1111875 | NR | Finnish and Swedish | Latent autoimmune diabetes in adults (hybrid of Type I and II) | GWAS |  | *[*[*7*](#_ENREF_7)*]* |
| rs5015480 | *NR* | *Mexican* | *Metabolic Syndrome* | *GWAS* | *Not associated with MS with T2D SNPs* | *[*[*8*](#_ENREF_8)*]* |
| Unable to source paper | *NR* | *Mexican* | *Metabolic Syndrome* | *GWAS* | *Not associated with MS with T2D SNPs* | *[*[*9*](#_ENREF_9)*]* |
| *rs1111875* | NR | *Chinese* | *PCOS* | *GWAS* | *No correlation* | *[*[*10*](#_ENREF_10)*]* |
| *rs1111875, rs5015480 and rs7923837* | NR | *Korean* | *PCOS* | *GWAS* | *No correlation (PCOS shares insulin resistance as a link to T2D).* | *[*[*11*](#_ENREF_11)*]* |
| *rs1111875 and rs7923837* | *rs10811661* | *Southern Indian Women* | *PCOS* | *GWAS* | *Not associated with PCOS* | *[*[*12*](#_ENREF_12)*]* |
| *rs1111875* | *rs10811661* | *Saudi Women* | *PCOS* | *GWAS* | *Not associated with PCOS* | *[*[*13*](#_ENREF_13)*]* |
| rs1111875 | rs10811661 | Korean | Post-transplant DM | GWAS |  | *[*[*14*](#_ENREF_14)*]* |
| rs1111875 | rs10811661 (p=0.07) | Caucasian Europeans | Reduced birth weight (when inherited) | GWAS |  | *[*[*15*](#_ENREF_15)*]* |
| *rs1111875 and rs7923837* | *rs2383207* | *Caucasian Children* | *Reduced birth weight (when inherited)* | *GWAS* | *Not associated with low birth weight* | *[*[*16*](#_ENREF_16)*]* |
| *rs1111875, rs5015480 and rs7923837* | *rs10811661 and rs564398* | *European* | *T1D* | *GWAS* | *Not associated with T1D* | *[*[*17*](#_ENREF_17)*]* |
| rs1111875 | rs10811661 | *British* | *T1D* | GWAS | *Not associated with T1D* | *[*[*18*](#_ENREF_18)*]* |
| *rs5015480* | NR | *German* | *T1D* | *GWAS* | *Was not related to T1D diabetes islet autoimmunity* | *[*[*19*](#_ENREF_19)*]* |
| rs1111875 | rs10811661 | Danish | T2D | GWAS |  | *[*[*20*](#_ENREF_20)*]* |
| rs1111875, rs5015480 and rs7923837 | rs10811661 | Japanese | T2D | GWAS |  | *[*[*21*](#_ENREF_21)*]* |
| rs1111875 | rs10811661 | Finnish and Swedish | T2D | GWAS |  | *[*[*22*](#_ENREF_22)*]* |
| rs1111875 | rs10811661 | Finnish | T2D | GWAS |  | *[*[*23*](#_ENREF_23)*]* |
| rs7923837 | NR | Canadian | T2D | GWAS | First identified? | *[*[*24*](#_ENREF_24)*]* |
| rs1111875 and rs5015480 | rs10811661 and rs564398 | British | T2D | GWAS |  | *[*[*25*](#_ENREF_25)*]* |
| rs1111875 | rs10811661 | French | T2D | GWAS | Noted mutations combine as risk factors | *[*[*26*](#_ENREF_26)*,* [*27*](#_ENREF_27)*]* |
| rs1111875 and rs7923837 | NR | Japanese | T2D | GWAS | Hhex-IDE-KIF111 | *[*[*28*](#_ENREF_28)*]* |
| rs1111875 | NR | German | T2D | GWAS |  | *[*[*29*](#_ENREF_29)*]* |
| rs1111875 | rs4402960 | Norwegian | T2D | GWAS |  | *[*[*30*](#_ENREF_30)*]* |
| rs1111875 | rs4402960 | Japanese | T2D | GWAS |  | *[*[*31*](#_ENREF_31)*]* |
| rs1111875 | rs10811661 | Korean | T2D | GWAS |  | *[*[*32*](#_ENREF_32)*]* |
| rs7923837 | rs10811661 | Korean/Hong Kong | T2D | GWAS |  | *[*[*33*](#_ENREF_33)*]* |
| rs1111875 | rs10811661 | Japanese | T2D | GWAS |  | *[*[*34*](#_ENREF_34)*]* |
| rs1111875 | rs10811661 | Northern Indian Sikh | T2D | GWAS |  | *[*[*35*](#_ENREF_35)*]* |
| rs1111875 and rs7923837 | NR | Dutch | T2D | GWAS |  | *[*[*36*](#_ENREF_36)*]* |
| rs5015480 (not rs1111875)* | rs10811661 | * Shanghai, but not Beijing / Han Chinese | T2D | GWAS |  | *[*[*37*](#_ENREF_37)*]* |
| *rs7923837* | NR | *Tunisian* | *T2D* | *GWAS* | *Not a risk factor for T2D* | *[*[*38*](#_ENREF_38)*]* |
| rs1111875, rs10509645 and rs10748582 | Not rs564398 | Han Chinese | T2D | GWAS |  | *[*[*39*](#_ENREF_39)*]* |
| rs7923837 | NR | Swedish | T2D | GWAS |  | *[*[*40*](#_ENREF_40)*]* |
| rs1111875 | NR | German | T2D | GWAS | Particularly related to impaired insulin secretion and insulin degradation. | *[*[*41*](#_ENREF_41)*]* |
| rs1111875, rs5015480 and rs7923837 | rs3731211, rs3731201, rs3217992, rs495490, rs523096, rs564398 and rs10811661 | Quebec, Canadian | T2D | GWAS | Associated with T2D susceptibility, via physiological changes including glucose intolerance, impaired insulin secretion or insulin resistance | *[*[*42*](#_ENREF_42)*]* |
| rs1111875 | NR | Finnish men | T2D | GWAS |  | *[*[*43*](#_ENREF_43)*]* |
| rs1111875 and rs7923837 | rs10811661 | Japanese | T2D | GWAS |  | *[*[*44*](#_ENREF_44)*]* |
| rs1111875 and rs7923837 | rs10811661 and rs564398 | Japanese | T2D | GWAS |  | *[*[*45*](#_ENREF_45)*]* |
| rs1111875 | rs10811661 | Indian | T2D | GWAS | Greater risk factor than that of Europeans | *[*[*46*](#_ENREF_46)*]* |
| rs7923837 | rs7020996 | Southern Indian | T2D | GWAS |  | *[*[*47*](#_ENREF_47)*]* |
| rs5015480 | rs10811661 | Han Chinese | T2D | GWAS |  | *[*[*48*](#_ENREF_48)*]* |
| rs1111875 | rs10811661 | Singaporean (Chinese/Malay/Indian) | T2D | GWAS |  | *[*[*49*](#_ENREF_49)*]* |
| rs1111875 | NR | Han Chinese | T2D | GWAS |  | *[*[*50*](#_ENREF_50)*]* |
| *rs1111875* | NR | *Chinese/East Asians (6 studies)* | *T2D* | *Meta-analysis* | *Not significant T2D risk factor* | *[*[*51*](#_ENREF_51)*]* |
| rs1111875 and rs7923837 | NR | Asian and Caucasian (22 studies) | T2D | Meta-analysis | (37K T2D patients vs 52K controls) and found ethnicity accounted for main source of heterogeneity and that these polymorphisms were conclusively risk factors for diabetes (especially in Asian and Caucasian populations). | *[*[*52*](#_ENREF_52)*]* |
| rs1111875 | rs10811661 | Pakistani | T2D | GWAS |  | *[*[*53*](#_ENREF_53)*]* |
| rs5015480 | NR | Korean women | T2D | GWAS |  | *[*[*54*](#_ENREF_54)*]* |
| rs1111875 | NR | Han Chinese (26 studies) | T2D | Meta-analysis | Variability also stratified with ethnicity | *[*[*55*](#_ENREF_55)*]* |
| rs7923837 | rs10811661 | Mexican | T2D | GWAS |  | *[*[*56*](#_ENREF_56)*]* |
| rs1111875 (49 studies), rs5015480 (18 studies) and rs7923837 (26 studies) | NR | All ethnic subclasses, especially Asian and Caucasian | T2D | Meta-analysis |  | *[*[*57*](#_ENREF_57)*]* |
| rs7923837 | NR | Han Chinese | T2D | GWAS | 10q23.33 contained genetic variants of IDE-KIF11-HHEX locus with rs7923837 being the strongest T2D susceptibility SNP | *[*[*58*](#_ENREF_58)*]* |
| rs1111875 | NR | Swedish | T2D | GWAS | Specifically affected insulin granule docking in beta islet cells from T2D patients | *[*[*59*](#_ENREF_59)*]* |
| rs1111875 and rs5015480 | NR | Indian (3 geographically distinct locations) | T2D | GWAS |  | *[*[*60*](#_ENREF_60)*]* |
| *rs7923837* | *rs10811661* | *Arabian* | *T2D* | *Meta-analysis* | *Not significant T2D risk factor* | *[*[*61*](#_ENREF_61)*]* |
| rs5015480 | rs10811661 | She Chinese | T2D | GWAS | Hhex is a risk factor in diabetic nephropathy and cardiovascular risk. Cdkn2a/b is associated as a risk factor for T2D and cardiovascular risk. | *[*[*62*](#_ENREF_62)*]* |
| NR | NR | Swedish | T2D | GWAS | Hhex SNPs in T2D associated with insulin secretion and glucagon secretion (and Cdkn2a/b in the former). | *[*[*63*](#_ENREF_63)*]* |
| rs1111875 | NR | Han Chinese (Taiwan) | T2D | GWAS | But differing allele frequency to European populations | *[*[*64*](#_ENREF_64)*]* |
| *rs11187146, rs5015480, rs4646957 and rs1111875* | NR | *Han Chinese* | *T2D* | *GWAS* | *Not a risk factor for T2D, T2D SNPs (rs7923837) associated with triglyceride levels and total cholesterol levels (rs2488075 and rs947591).* | *[*[*65*](#_ENREF_65)*]* |
| rs1111875 | rs10811661 | *Omani* | *T2D* | *GWAS* | *Hhex SNP not a risk factor for T2D, but Cdkn2a/b T2D SNPs were associated with increased T2D risk.* | *[*[*66*](#_ENREF_66)*]* |
| rs1111875 | NR | *Sudanese* | T2D | GWAS |  | *[*[*67*](#_ENREF_67)*]* |
| rs7923837 | NR | Han Chinese | T2D | GWAS |  | *[*[*68*](#_ENREF_68)*]* |
| rs5015480 | NR | Greek Cypriot | T2D | GWAS |  | *[*[*69*](#_ENREF_69)*]* |
| rs1111875 | rs10811661 | Thai | T2D | GWAS | Hhex SNP not a risk factor for T2D (but trending), but Cdkn2a/b T2D SNPs were associated with increased T2D risk. | *[*[*70*](#_ENREF_70)*]* |
| rs5015480 | NR | Heavy smoking Korean men | T2D | GWAS |  | *[*[*71*](#_ENREF_71)*]* |
| rs1111875 and rs5015480 (but not rs7923837) | NR | Iranian | T2D | GWAS |  | *[*[*72*](#_ENREF_72)*]* |
| rs1111875 | NR | Mexican | T2D | GWAS |  | *[*[*73*](#_ENREF_73)*]* |
| rs1977833 | ND | Emiratis | T2D | GWAS | This SNP was included as a proxy for rs1111875. | *[*[*74*](#_ENREF_74)*]* |
| rs1111875 and rs5015480 | ND | Han Chinese | T2D | GWAS |  | *[*[*75*](#_ENREF_75)*]* |
| rs1111875 | NR | Bangladeshi | T2D | GWAS | *Hhex not associated with CVD.* | *[*[*76*](#_ENREF_76)*]* |
| rs1111875 | NR | Hawaiian women, and in particular, Pacific Islander and Filipino populations | T2D | GWAS |  | *[*[*77*](#_ENREF_77)*]* |
| *rs1111875* | *rs7018475* | *Brazilian* | *T2D* | *GWAS* | *Hhex SNP not a risk factor for T2D, but Cdkn2a/b T2D SNPs were associated with increased T2D risk (in non-smokers/women).* | *[*[*78*](#_ENREF_78)*]* |
| rs1111875 | ND | Danish | T2D | GWAS |  | *[*[*79*](#_ENREF_79)*]* |
| *rs1111875* | *rs10811661* | *Indian (Uttarakhand subpopulation)* | *T2D* | *GWAS* | *Hhex was not a significant T2D risk factor (p=0.138), but Cdkn2a/b was a significant risk factor.* | *[*[*80*](#_ENREF_80)*]* |
| rs7923837 | NR | Saudis | T2D/overweight | GWAS |  | *[*[*81*](#_ENREF_81)*]* |
| rs5015480 | NR | Han Chinese | T2D/famine pre-exposure | GWAS | The effect of Hhex SNP rs5015480 was reduced following pre-exposure to famine. | *[*[*82*](#_ENREF_82)*]* |
| rs1111875 | NR | Han Chinese | T2D - Diabetic retinopathy in T2D patients | GWAS |  | *[*[*83*](#_ENREF_83)*]* |
| rs290481 | NR | *Han Chinese* | T2D - Increased cancer risk in T2D | GWAS |  | *[*[*84*](#_ENREF_84)*]* |
| rs1111875 | rs10811661 | Non-diabetic Finnish men | T2D - Increased hepatic insulin sensitivity | GWAS | Also associated with impaired insulin secretion, was also associated with increased hepatic insulin sensitivity | *[*[*85*](#_ENREF_85)*]* |
| rs1111875 | NR | European | T2D - Reduced pancreatic beta-cell function | GWAS |  | *[*[*86*](#_ENREF_86)*]* |
| *rs1111875* | *rs10811661* | *Han Chinese* | *T2D and low birth weight* | *GWAS* | *Not associated with T2D and low birth weight* | *[*[*87*](#_ENREF_87)*]* |
| rs1111875 | rs10811661 | Italian | T2D and low birth weight | GWAS |  | *[*[*88*](#_ENREF_88)*]* |
| rs5015480 and rs10882102 | NR | German | T2D and low birth weight in mothers with T1D | GWAS | Also associated with low birth weight in mothers with T1D | *[*[*89*](#_ENREF_89)*]* |
| rs1111875 | rs10811661 | Han Chinese | T2D and Metabolic Syndrome | GWAS |  | *[*[*90*](#_ENREF_90)*]* |
| rs1111875 | rs10811661 | Han Chinese | T2D and Fasting Plasma Glucose | GWAS |  | *[*[*91*](#_ENREF_91)*]* |
| rs1111875 and rs7923837 | NR | German | T2D in association with reduced insulin secretion | GWAS |  | *[*[*92*](#_ENREF_92)*,* [*93*](#_ENREF_93)*]* |
| rs1111875 | rs7020996 | South Indian | Early Onset T2D | GWAS |  | *[*[*94*](#_ENREF_94)*]* |

*Studies lacking an association with the Hhex and/or CDKN2A SNPs are italicised throughout. Studies ordered in terms of disease, then year of publication, then alphabetical first author surname.*

Abbreviations: CVD, Cardio-Vascular Disease, GDM, Gestational Diabetes Myelitis, GWAS, Genome-Wide Association Study, MS, Metabolic Syndrome, NR, Not Reported, PCOS, Poly-Cystic Ovary Syndrome, SNP, Single Nucleotide Polymorphism, T1D, Type I Diabetes, T2D, Type II Diabetes.

References:

1. Cho, Y.M., et al., *Type 2 diabetes-associated genetic variants discovered in the recent genome-wide association studies are related to gestational diabetes mellitus in the Korean population.* Diabetologia, 2009. **52**(2): p. 253-61.

2. Ekelund, M., et al., *Genetic prediction of postpartum diabetes in women with gestational diabetes mellitus.* Diabetes Res Clin Pract, 2012. **97**(3): p. 394-8.

3. Bysani, M., et al., *ATAC-seq reveals alterations in open chromatin in pancreatic islets from subjects with type 2 diabetes.* Sci Rep, 2019. **9**(1): p. 7785.

4. Tarnowski, M., et al., *Hematopoietically expressed homeobox (HHEX) gene polymorphism (rs5015480) is associated with increased risk of gestational diabetes mellitus.* Clin Genet, 2017. **91**(6): p. 843-848.

5. Wang, X., et al., *The association between HHEX single-nucleotide polymorphism rs5015480 and gestational diabetes mellitus: A meta-analysis.* Medicine (Baltimore), 2020. **99**(12): p. e19478.

6. Kwak, S.H., et al., *Clinical and genetic risk factors for type 2 diabetes at early or late post partum after gestational diabetes mellitus.* J Clin Endocrinol Metab, 2013. **98**(4): p. E744-52.

7. Andersen, M.K., et al., *Type 2 diabetes susceptibility gene variants predispose to adult-onset autoimmune diabetes.* Diabetologia, 2014. **57**(9): p. 1859-68.

8. Cruz, M., et al., *Candidate gene association study conditioning on individual ancestry in patients with type 2 diabetes and metabolic syndrome from Mexico City.* Diabetes Metab Res Rev, 2010. **26**(4): p. 261-70.

9. Gutiérrez-Vidal, R., et al., *LOC387761 polymorphism is associated with type 2 diabetes in the Mexican population.* Genet Test Mol Biomarkers, 2011. **15**(1-2): p. 79-83.

10. Xu, P., et al., *Polymorphisms of TCF7L2 and HHEX genes in Chinese women with polycystic ovary syndrome.* J Assist Reprod Genet, 2010. **27**(1): p. 23-8.

11. Kim, J.J., et al., *Polycystic ovary syndrome is not associated with polymorphisms of the TCF7L2, CDKAL1, HHEX, KCNJ11, FTO and SLC30A8 genes.* Clin Endocrinol (Oxf), 2012. **77**(3): p. 439-45.

12. Reddy, B.M., et al., *Association of type 2 diabetes mellitus genes in polycystic ovary syndrome aetiology among women from southern India.* Indian J Med Res, 2016. **144**(3): p. 400-408.

13. Ezzidi, I., et al., *Impact of variants on type-2 diabetes risk genes identified through genomewide association studies in polycystic ovary syndrome: a case–control study.* Journal of Genetics, 2018. **97**(5): p. 1213-1223.

14. Kang, E.S., et al., *Association of common type 2 diabetes risk gene variants and posttransplantation diabetes mellitus in renal allograft recipients in Korea.* Transplantation, 2009. **88**(5): p. 693-8.

15. Freathy, R.M., et al., *Type 2 diabetes risk alleles are associated with reduced size at birth.* Diabetes, 2009. **58**(6): p. 1428-33.

16. Zhao, J., et al., *Examination of type 2 diabetes loci implicates CDKAL1 as a birth weight gene.* Diabetes, 2009. **58**(10): p. 2414-8.

17. Qu, H.Q., et al., *Association analysis of type 2 diabetes Loci in type 1 diabetes.* Diabetes, 2008. **57**(7): p. 1983-6.

18. Raj, S.M., et al., *No association of multiple type 2 diabetes loci with type 1 diabetes.* Diabetologia, 2009. **52**(10): p. 2109-16.

19. Winkler, C., et al., *Lack of association of type 2 diabetes susceptibility genotypes and body weight on the development of islet autoimmunity and type 1 diabetes.* PLoS One, 2012. **7**(4): p. e35410.

20. Grarup, N., et al., *Studies of association of variants near the HHEX, CDKN2A/B, and IGF2BP2 genes with type 2 diabetes and impaired insulin release in 10,705 Danish subjects: validation and extension of genome-wide association studies.* Diabetes, 2007. **56**(12): p. 3105-11.

21. Horikoshi, M., et al., *Variations in the HHEX gene are associated with increased risk of type 2 diabetes in the Japanese population.* Diabetologia, 2007. **50**(12): p. 2461-6.

22. Saxena, R., et al., *Genome-wide association analysis identifies loci for type 2 diabetes and triglyceride levels.* Science, 2007. **316**(5829): p. 1331-6.

23. Scott, L.J., et al., *A genome-wide association study of type 2 diabetes in Finns detects multiple susceptibility variants.* Science, 2007. **316**(5829): p. 1341-5.

24. Sladek, R., et al., *A genome-wide association study identifies novel risk loci for type 2 diabetes.* Nature, 2007. **445**(7130): p. 881-5.

25. Zeggini, E., et al., *Replication of genome-wide association signals in UK samples reveals risk loci for type 2 diabetes.* Science, 2007. **316**(5829): p. 1336-41.

26. Cauchi, S., et al., *Post genome-wide association studies of novel genes associated with type 2 diabetes show gene-gene interaction and high predictive value.* PLoS One, 2008. **3**(5): p. e2031.

27. Cauchi, S., et al., *Analysis of novel risk loci for type 2 diabetes in a general French population: the D.E.S.I.R. study.* J Mol Med (Berl), 2008. **86**(3): p. 341-8.

28. Furukawa, Y., et al., *Polymorphisms in the IDE-KIF11-HHEX gene locus are reproducibly associated with type 2 diabetes in a Japanese population.* J Clin Endocrinol Metab, 2008. **93**(1): p. 310-4.

29. Herder, C., et al., *Variants of the PPARG, IGF2BP2, CDKAL1, HHEX, and TCF7L2 genes confer risk of type 2 diabetes independently of BMI in the German KORA studies.* Horm Metab Res, 2008. **40**(10): p. 722-6.

30. Hertel, J.K., et al., *Genetic analysis of recently identified type 2 diabetes loci in 1,638 unselected patients with type 2 diabetes and 1,858 control participants from a Norwegian population-based cohort (the HUNT study).* Diabetologia, 2008. **51**(6): p. 971-7.

31. Horikawa, Y., et al., *Replication of genome-wide association studies of type 2 diabetes susceptibility in Japan.* J Clin Endocrinol Metab, 2008. **93**(8): p. 3136-41.

32. Lee, Y.H., et al., *Association between polymorphisms in SLC30A8, HHEX, CDKN2A/B, IGF2BP2, FTO, WFS1, CDKAL1, KCNQ1 and type 2 diabetes in the Korean population.* J Hum Genet, 2008. **53**(11-12): p. 991-998.

33. Ng, M.C., et al., *Implication of genetic variants near TCF7L2, SLC30A8, HHEX, CDKAL1, CDKN2A/B, IGF2BP2, and FTO in type 2 diabetes and obesity in 6,719 Asians.* Diabetes, 2008. **57**(8): p. 2226-33.

34. Omori, S., et al., *Association of CDKAL1, IGF2BP2, CDKN2A/B, HHEX, SLC30A8, and KCNJ11 with susceptibility to type 2 diabetes in a Japanese population.* Diabetes, 2008. **57**(3): p. 791-5.

35. Sanghera, D.K., et al., *Impact of nine common type 2 diabetes risk polymorphisms in Asian Indian Sikhs: PPARG2 (Pro12Ala), IGF2BP2, TCF7L2 and FTO variants confer a significant risk.* BMC Med Genet, 2008. **9**: p. 59.

36. van Vliet-Ostaptchouk, J.V., et al., *HHEX gene polymorphisms are associated with type 2 diabetes in the Dutch Breda cohort.* Eur J Hum Genet, 2008. **16**(5): p. 652-6.

37. Wu, Y., et al., *Common variants in CDKAL1, CDKN2A/B, IGF2BP2, SLC30A8, and HHEX/IDE genes are associated with type 2 diabetes and impaired fasting glucose in a Chinese Han population.* Diabetes, 2008. **57**(10): p. 2834-42.

38. Ezzidi, I., et al., *Contribution of type 2 diabetes associated loci in the Arabic population from Tunisia: a case-control study.* BMC Med Genet, 2009. **10**: p. 33.

39. Hu, C., et al., *PPARG, KCNJ11, CDKAL1, CDKN2A-CDKN2B, IDE-KIF11-HHEX, IGF2BP2 and SLC30A8 are associated with type 2 diabetes in a Chinese population.* PLoS One, 2009. **4**(10): p. e7643.

40. Nordman, S., et al., *Loci of TCF7L2, HHEX and IDE on chromosome 10q and the susceptibility of their genetic polymorphisms to type 2 diabetes.* Exp Clin Endocrinol Diabetes, 2009. **117**(4): p. 186-90.

41. Pivovarova, O., et al., *The influence of genetic variations in HHEX gene on insulin metabolism in the German MESYBEPO cohort.* Diabetes Metab Res Rev, 2009. **25**(2): p. 156-62.

42. Ruchat, S.M., et al., *Association between insulin secretion, insulin sensitivity and type 2 diabetes susceptibility variants identified in genome-wide association studies.* Acta Diabetol, 2009. **46**(3): p. 217-26.

43. Stancakova, A., et al., *Association of 18 confirmed susceptibility loci for type 2 diabetes with indices of insulin release, proinsulin conversion, and insulin sensitivity in 5,327 nondiabetic Finnish men.* Diabetes, 2009. **58**(9): p. 2129-36.

44. Tabara, Y., et al., *Replication study of candidate genes associated with type 2 diabetes based on genome-wide screening.* Diabetes, 2009. **58**(2): p. 493-8.

45. Takeuchi, F., et al., *Confirmation of multiple risk Loci and genetic impacts by a genome-wide association study of type 2 diabetes in the Japanese population.* Diabetes, 2009. **58**(7): p. 1690-9.

46. Chauhan, G., et al., *Impact of common variants of PPARG, KCNJ11, TCF7L2, SLC30A8, HHEX, CDKN2A, IGF2BP2, and CDKAL1 on the risk of type 2 diabetes in 5,164 Indians.* Diabetes, 2010. **59**(8): p. 2068-74.

47. Chidambaram, M., V. Radha, and V. Mohan, *Replication of recently described type 2 diabetes gene variants in a South Indian population.* Metabolism, 2010. **59**(12): p. 1760-6.

48. Han, X., et al., *Implication of genetic variants near SLC30A8, HHEX, CDKAL1, CDKN2A/B, IGF2BP2, FTO, TCF2, KCNQ1, and WFS1 in type 2 diabetes in a Chinese population.* BMC Med Genet, 2010. **11**: p. 81.

49. Tan, J.T., et al., *Polymorphisms identified through genome-wide association studies and their associations with type 2 diabetes in Chinese, Malays, and Asian-Indians in Singapore.* J Clin Endocrinol Metab, 2010. **95**(1): p. 390-7.

50. Zhao, J., et al., *Examination of all type 2 diabetes GWAS loci reveals HHEX-IDE as a locus influencing pediatric BMI.* Diabetes, 2010. **59**(3): p. 751-5.

51. Zhou, D.Z., et al., *Variations in/nearby genes coding for JAZF1, TSPAN8/LGR5 and HHEX-IDE and risk of type 2 diabetes in Han Chinese.* J Hum Genet, 2010. **55**(12): p. 810-5.

52. Cai, Y., et al., *Meta-analysis of the effect of HHEX gene polymorphism on the risk of type 2 diabetes.* Mutagenesis, 2011. **26**(2): p. 309-14.

53. Rees, S.D., et al., *Replication of 13 genome-wide association (GWA)-validated risk variants for type 2 diabetes in Pakistani populations.* Diabetologia, 2011. **54**(6): p. 1368-74.

54. Ryoo, H., et al., *Heterogeneity of genetic associations of CDKAL1 and HHEX with susceptibility of type 2 diabetes mellitus by gender.* Eur J Hum Genet, 2011. **19**(6): p. 672-5.

55. Wang, Y., et al., *Quantitative assessment of the influence of hematopoietically expressed homeobox variant (rs1111875) on type 2 diabetes risk.* Mol Genet Metab, 2011. **102**(2): p. 194-9.

56. Gamboa-Melendez, M.A., et al., *Contribution of common genetic variation to the risk of type 2 diabetes in the Mexican Mestizo population.* Diabetes, 2012. **61**(12): p. 3314-21.

57. Li, X., et al., *Hematopoietically-expressed homeobox gene three widely-evaluated polymorphisms and risk for diabetes: a meta-analysis.* PLoS One, 2012. **7**(11): p. e49917.

58. Qian, Y., et al., *Genetic variants of IDE-KIF11-HHEX at 10q23.33 associated with type 2 diabetes risk: a fine-mapping study in Chinese population.* PLoS One, 2012. **7**(4): p. e35060.

59. Rosengren, A.H., et al., *Reduced insulin exocytosis in human pancreatic beta-cells with gene variants linked to type 2 diabetes.* Diabetes, 2012. **61**(7): p. 1726-33.

60. Ali, S., et al., *Replication of type 2 diabetes candidate genes variations in three geographically unrelated Indian population groups.* PLoS One, 2013. **8**(3): p. e58881.

61. Al-Rubeaan, K., et al., *ACE I/D and MTHFR C677T polymorphisms are significantly associated with type 2 diabetes in Arab ethnicity: a meta-analysis.* Gene, 2013. **520**(2): p. 166-77.

62. Chen, G., et al., *Association study of genetic variants of 17 diabetes-related genes/loci and cardiovascular risk and diabetic nephropathy in the Chinese She population.* J Diabetes, 2013. **5**(2): p. 136-45.

63. Jonsson, A., et al., *Effects of common genetic variants associated with type 2 diabetes and glycemic traits on alpha- and beta-cell function and insulin action in humans.* Diabetes, 2013. **62**(8): p. 2978-83.

64. Chang, Y.C., et al., *Validation of type 2 diabetes risk variants identified by genome-wide association studies in Han Chinese population: a replication study and meta-analysis.* PLoS One, 2014. **9**(4): p. e95045.

65. Liu, S., et al., *Genetic variants at 10q23.33 are associated with plasma lipid levels in a Chinese population.* J Biomed Res, 2014. **28**(1): p. 53-8.

66. Al-Sinani, S., et al., *Association of gene variants with susceptibility to type 2 diabetes among Omanis.* World J Diabetes, 2015. **6**(2): p. 358-66.

67. Ibrahim, A.T., et al., *Candidate gene analysis supports a role for polymorphisms at TCF7L2 as risk factors for type 2 diabetes in Sudan.* J Diabetes Metab Disord, 2015. **15**: p. 4.

68. Wei, F.J., et al., *Quantitative candidate gene association studies of metabolic traits in Han Chinese type 2 diabetes patients.* Genet Mol Res, 2015. **14**(4): p. 15471-81.

69. Votsi, C., et al., *Type 2 Diabetes Susceptibility in the Greek-Cypriot Population: Replication of Associations with TCF7L2, FTO, HHEX, SLC30A8 and IGF2BP2 Polymorphisms.* Genes (Basel), 2017. **8**(1).

70. Plengvidhya, N., et al., *Impact of KCNQ1, CDKN2A/2B, CDKAL1, HHEX, MTNR1B, SLC30A8, TCF7L2, and UBE2E2 on risk of developing type 2 diabetes in Thai population.* BMC Med Genet, 2018. **19**(1): p. 93.

71. Sull, J.W., T.Y. Lee, and S.H. Jee, *Effect of smoking on the association of HHEX (rs5015480) with diabetes among Korean women and heavy smoking men.* BMC Med Genet, 2018. **19**(1): p. 68.

72. Galavi, H., et al., *HHEX gene polymorphisms and type 2 diabetes mellitus: A case-control report from Iran.* J Cell Biochem, 2019. **120**(10): p. 16445-16451.

73. Dominguez-Cruz, M.G., et al., *Maya gene variants related to the risk of type 2 diabetes in a family-based association study.* Gene, 2020. **730**: p. 144259.

74. Osman, W., et al., *Genetics of type 2 diabetes and coronary artery disease and their associations with twelve cardiometabolic traits in the United Arab Emirates population.* Gene, 2020. **750**: p. 144722.

75. Li, C., et al., *Association Between Single Nucleotide Polymorphisms in CDKAL1 and HHEX and Type 2 Diabetes in Chinese Population.* Diabetes Metab Syndr Obes, 2020. **13**: p. 5113-5123.

76. Aka, T.D., et al., *Risk of type 2 diabetes mellitus and cardiovascular complications in KCNJ11, HHEX and SLC30A8 genetic polymorphisms carriers: A case-control study.* Heliyon, 2021. **7**(11): p. e08376.

77. Benny, P., et al., *Genetic risk factors associated with gestational diabetes in a multi-ethnic population.* PLoS One, 2021. **16**(12): p. e0261137.

78. Cirelli, T., et al., *Association of type 2 diabetes mellitus and periodontal disease susceptibility with genome-wide association-identified risk variants in a Southeastern Brazilian population.* Clin Oral Investig, 2021. **25**(6): p. 3873-3892.

79. Deshmukh, H.A., et al., *Genome-Wide Association Analysis of Pancreatic Beta-Cell Glucose Sensitivity.* J Clin Endocrinol Metab, 2021. **106**(1): p. 80-90.

80. Verma, A.K., et al., *Association Between CDKAL1, HHEX, CDKN2A/2B and IGF2BP2 Gene Polymorphisms and Susceptibility to Type 2 Diabetes in Uttarakhand, India.* Diabetes Metab Syndr Obes, 2021. **14**: p. 23-36.

81. Alfaifi, M., *Contribution of genetic variant identified in HHEX gene in the overweight Saudi patients confirmed with type 2 diabetes mellitus.* Saudi J Biol Sci, 2022. **29**(2): p. 804-808.

82. Song, C., et al., *Ten SNPs May Affect Type 2 Diabetes Risk in Interaction with Prenatal Exposure to Chinese Famine.* Nutrients, 2020. **12**(12).

83. Peng, D., et al., *CDKAL1 rs7756992 is associated with diabetic retinopathy in a Chinese population with type 2 diabetes.* Sci Rep, 2017. **7**(1): p. 8812.

84. Ma, R.C., et al., *Genetic variants for type 2 diabetes and new-onset cancer in Chinese with type 2 diabetes.* Diabetes Res Clin Pract, 2014. **103**(2): p. 328-37.

85. Vangipurapu, J., et al., *Association of indices of liver and adipocyte insulin resistance with 19 confirmed susceptibility loci for type 2 diabetes in 6,733 non-diabetic Finnish men.* Diabetologia, 2011. **54**(3): p. 563-71.

86. Pascoe, L., et al., *Common variants of the novel type 2 diabetes genes CDKAL1 and HHEX/IDE are associated with decreased pancreatic beta-cell function.* Diabetes, 2007. **56**(12): p. 3101-4.

87. Sun, X.F., et al., *Positive Association Between Type 2 Diabetes Risk Alleles Near CDKAL1 and Reduced Birthweight in Chinese Han Individuals.* Chin Med J (Engl), 2015. **128**(14): p. 1873-8.

88. Pulizzi, N., et al., *Interaction between prenatal growth and high-risk genotypes in the development of type 2 diabetes.* Diabetologia, 2009. **52**(5): p. 825-9.

89. Winkler, C., et al., *HHEX-IDE polymorphism is associated with low birth weight in offspring with a family history of type 1 diabetes.* J Clin Endocrinol Metab, 2009. **94**(10): p. 4113-5.

90. Kong, X., et al., *The Association of Type 2 Diabetes Loci Identified in Genome-Wide Association Studies with Metabolic Syndrome and Its Components in a Chinese Population with Type 2 Diabetes.* PLoS One, 2015. **10**(11): p. e0143607.

91. Zhao, Q., et al., *Cross-sectional and longitudinal replication analyses of genome-wide association loci of type 2 diabetes in Han Chinese.* PLoS One, 2014. **9**(3): p. e91790.

92. Staiger, H., et al., *Polymorphisms within novel risk loci for type 2 diabetes determine beta-cell function.* PLoS One, 2007. **2**(9): p. e832.

93. Staiger, H., et al., *A candidate type 2 diabetes polymorphism near the HHEX locus affects acute glucose-stimulated insulin release in European populations: results from the EUGENE2 study.* Diabetes, 2008. **57**(2): p. 514-7.

94. Liju, S., et al., *Impact of type 2 diabetes variants identified through genome-wide association studies in early-onset type 2 diabetes from South Indian population.* Genomics Inform, 2020. **18**(3): p. e27.
